# ERK1/2 signalling dynamics promote neural differentiation by regulating chromatin accessibility and the polycomb repressive complex

**DOI:** 10.1371/journal.pbio.3000221

**Published:** 2022-12-01

**Authors:** Claudia I. Semprich, Lindsay Davidson, Adriana Amorim Torres, Harshil Patel, James Briscoe, Vicki Metzis, Kate G. Storey

**Affiliations:** 1 Division of Cell & Developmental Biology, School of Life Sciences, University of Dundee, Scotland, United Kingdom; 2 The Francis Crick Institute, London, United Kingdom; 3 Institute of Clinical Sciences, Faculty of Medicine, Imperial College London, London, United Kingdom; California Institute of Technology, UNITED STATES

## Abstract

Fibroblast growth factor (FGF) is a neural inducer in many vertebrate embryos, but how it regulates chromatin organization to coordinate the activation of neural genes is unclear. Moreover, for differentiation to progress, FGF signalling must decline. Why these signalling dynamics are required has not been determined. Here, we show that dephosphorylation of the FGF effector kinase ERK1/2 rapidly increases chromatin accessibility at neural genes in mouse embryos, and, using ATAC-seq in human embryonic stem cell derived spinal cord precursors, we demonstrate that this occurs genome-wide across neural genes. Importantly, ERK1/2 inhibition induces precocious neural gene transcription, and this involves dissociation of the polycomb repressive complex from key gene loci. This takes place independently of subsequent loss of the repressive histone mark H3K27me3 and transcriptional onset. Transient ERK1/2 inhibition is sufficient for the dissociation of the repressive complex, and this is not reversed on resumption of ERK1/2 signalling. Moreover, genomic footprinting of sites identified by ATAC-seq together with ChIP-seq for polycomb protein Ring1B revealed that ERK1/2 inhibition promotes the occupancy of neural transcription factors (TFs) at non-polycomb as well as polycomb associated sites. Together, these findings indicate that ERK1/2 signalling decline promotes global changes in chromatin accessibility and TF binding at neural genes by directing polycomb and other regulators and appears to serve as a gating mechanism that provides directionality to the process of differentiation.

## Abstract 2

FGF is a neural inducer in many vertebrate embryos, but does it regulates chromatin organization to coordinate the activation of neural genes? This study reveals how a decline in FGF effector kinase ERK1/2 signaling promotes neural differentiation by regulating chromatin accessibility and polycomb protein occupancy at neural genes in the mouse embryo and in human pluripotent cell-derived neural precursors.

## Introduction

The identities of signals that induce particular cell fates are now well established, but how such signalling regulates chromatin to coordinate the transcription of differentiation genes and engage a differentiation programme are not well understood. Fibroblast growth factor (FGF) signalling has been implicated in the acquisition of neural cell fate in many vertebrate embryos ([1–7]; reviewed in [8]) (although the timing of involvement varies between species and anteroposterior regions; [6,9,10]). Intriguingly, while FGF is required for neural induction in most of these contexts, its decline is also necessary for differentiation progression.

**Fig 1:**
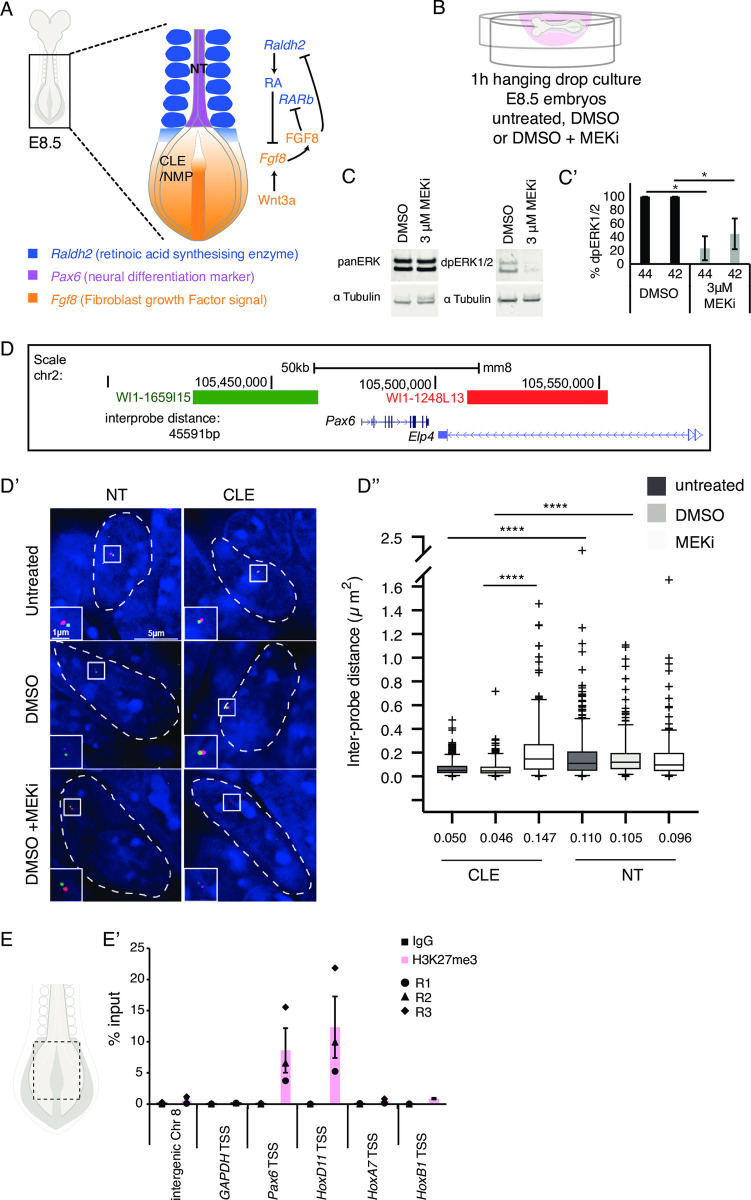
Rapid chromatin decompaction at the neural progenitor gene *Pax6* in the caudal lateral epiblast following ERK1/2 dephosphorylation in the developing mouse embryo. (**A**) E8.5 mouse embryo schematic, showing the caudal region and signalling pathways known to regulate neural differentiation. (**B**) Schematic of the hanging drop culture used to treat E8.5 mouse embryos with small molecule MEKi (PD184352) or vehicle control DMSO for 1 hour. (**C** and **C’**) Representative western blot of embryo lysates probed with antibodies against total (panERK1/2) and phosphorylated ERK1/2 (dpERK1/2) and LiCOR quantification data (*n* = 3 independent experiments, error bar = SEM, * *p* = <0.05) (S1 Data). (**D**) Position of fosmid probes flanking the *Pax6* locus (interprobe probe distance ca. 45 kb). (**D’**) Representative examples of fosmid probes in individual NT and CLE nuclei (white dashed line) visualised with DAPI (blue) in each untreated condition, vehicle control (DMSO) and MEKi (in DMSO) treated. (**D”**) Interprobe distance measured in >50 nuclei in NT and CLE in each of three embryos in each condition (*n* = > 150 nuclei/condition/region, Mann–Whitney test/rank-sum test **** *p* ≤ 0.0001) (S1 Data). (**E**) Schematic showing caudal end explant (full tissue thickness taken) in E8.5 embryo. (**E’**) Chromatin from three biological replicates, each consisting of 30 pooled explants, were interrogated for H3K27me3 levels at TSSs of *Pax6*, known PRC target *HoxD11* and at control regions (see text) compared to IgG background. Three biological replicates (circle, triangle, and diamond) and average (bar) shown, error bar = SEM (S1 Data). CLE, caudal lateral epiblast; FGF, fibroblast growth factor; IgG, immunoglobulin G; MEKi, MEK inhibitor; NT, neural tube; PRC, polycomb repressive complex; RA, retinoic acid; Raldh2, retinaldehyde dehydrogenase 2; RAR, retinoic acid receptor; TSS, transcription start site.

The requirement for transient FGF signalling to promote neural differentiation is particularly evident in the elongating embryonic body axis in which the spinal cord is generated progressively. Here, there is a clear spatial separation of the temporal events of differentiation. FGF acts, along with Wnt signalling, in the caudal lateral epiblast (CLE)/node streak border (which later form the tailbud) to maintain a bipotent cell population known as neuromesodermal progenitors (NMPs), which progressively gives rise to the spinal cord and paraxial mesoderm (Fig 1A) [11–19]. Blocking FGF signalling in this cell population accelerates the onset of neural differentiation genes, and ectopic maintenance of FGF inhibits this step [20–22]. During normal development, differentiation onset is promoted by rising retinoid signalling, provided by adjacent paraxial mesoderm, which represses *Fgf8*, restricting it to the tail end (Fig 1A) ([21–24]; reviewed in [25]). These findings indicate that a decline in FGF signalling promotes neural differentiation; however, little is known about the mechanism(s) by which such signalling dynamics mediate the coordinated activation of neural differentiation genes.

As NMP progeny leave the CLE and embark on neural differentiation, they experience loss of the FGF effector kinase ERK1/2/MAPK (hereafter referred to as ERK1/2) activity in both chick and mouse embryos [26,27]. This suggests that ERK1/2 signalling dynamics may regulate the onset of neural differentiation. The in vitro manipulation of embryonic stem (ES) cells has provided some insight into the involvement of ERK1/2 signalling in this process. Rather than promoting differentiation, however, inhibition of ERK1/2 activity in mouse ES cells supports pluripotency [28,29], and exposure to FGF/ERK1/2 signalling appears to be an initial step in differentiation. In agreement with this, FGF is required for neural differentiation of mES cells [30–32], although the timing of this requirement differs between assays [33]. Importantly, however, only a short critical period of ERK1/2 signalling is required in mES cells for subsequent expression of neural genes [31], after which FGF inhibition then accelerates neural differentiation [31,34,35]. Moreover, in mouse epiblast stem cells (mEpiSCs), which rely on FGF for self-renewal, prolonged FGF signalling abrogates neural differentiation [36]. Consistent with this in both mEpiSCs and human ES cells (which also depend on FGF for self-renewal), inhibition of FGF/ERK1/2 signalling promotes neural differentiation [34,36]. These findings indicate that temporal control over FGF/ERK1/2 signalling is instrumental in the establishment of neural identity from epiblast cell precursors.

A clue to the mechanism by which FGF regulates neural differentiation was revealed by analysis of chromatin organisation at key neural progenitor genes in the mouse embryo CLE [37]. This showed using fluorescent in situ hybridisation (FISH) that inhibition of FGF signalling in embryos decompacts chromatin around neural genes such as *Pax6*. This increased chromatin accessibility was also found in *Raldh2* mutant embryos exposed to FGFR inhibitor in which retinoid deficiency resulted in the failure of *Pax6* expression [37]. This indicated that FGF regulation of chromatin organisation is molecularly distinct from the machinery that drives subsequent gene transcription but left open the question of how FGF signalling modifies chromatin organisation.

Potential targets for FGF signalling include the polycomb repressive complexes 1 and 2 (PRC1 and 2), which cooperate in the repression of differentiation genes in many contexts ([38–44]; reviewed in [45]). PRC2 is composed of core subunits Ezh1 or Ezh2, Eed, Suz12, and associated context specific accessory proteins Rbbp4 or Rbbp7, Mtf2, Jarid2, and polycomb-like proteins Pali1/2 (isoforms of Lcor/Lcorl), Epop, and Aebp2 [46–50] and, via Ezh2, catalyses the methylation of histone 3 lysine 27 (H3K27me2/3) [46,51–54], which in mES cells is augmented by Jarid2 [55–59]. PRC1 contains the E3 ubiquitin ligase, Ring1A or B that catalyses mono-ubiquitination on lysine 119 of histone 2A (H2AK119Ub) and, in its canonical form, includes (cPRC1), PCGF2 (MEL18) or PCGF4 (BMI1), and a PHC (PHC1,2 or 3) and CBX subunit (CBX2, 4, 6, 7, or 8); PRC1 also has varied noncanonical (ncPRC1) forms (reviewed in [60]). Early studies established that PRC2 and PRC1 cooperate to recruit the composite complex: methylation of H3K27 provides a docking site for PRC1 [61,62], and, in turn, PRC1-dependent H2A ubiquitylation provides a scaffold for PRC2 binding [59,63,64], while ncPRC1 includes PRC2-independent PRC1 recruitment and can be involved in context-specific gene repression or activation [60,65,66]. Cooperative PRC2 and PRC1 interactions create polycomb domains characterised by gene repressive histone marks H3K27me3 and H2AK119Ub. PRC1-generated chromatin compaction has been elucidated in mESCs [67–70], and such changes in chromatin organisation are mediated by a range of mechanisms that include nucleosome compaction [71] that in mammals involves CBX2 [72], formation of Polycomb Group (PcG) bodies and higher-order subnuclear clustering via PHC2 [60,73].

The PRC1 core protein Ring1B is required for gastrulation in the mouse embryo [74] (and see [75]), and conditional Ring1B loss in motor neuron precursors has demonstrated its further requirement for maintenance of neuronal subtype identities [76], while roles of H2AK119Ub-dependent and H2AK119Ub-independent PRC1 activity have been identified in the developing cortex at early and late stages, respectively [77]. Ring1B is also present in both canonical and noncanonical PRC1 complexes [78]. Proteomic analyses further show dynamic changes in PRC complexes and subcomplexes during mESC neural differentiation [46] that prefigure a further and later role for PRC1 in augmenting enhancer–promoter interactions during gene activation [66,79,80]. Intriguingly, ERK1/2 inhibition in mES cells, while driving cells into a naïve pluripotent cell state, leads to reduction in PRC2 at developmental genes [81–83]. This suggests a role for ERK1/2 signalling in the recruitment or maintenance of polycomb-mediated repression. There is also evidence that this may involve regulation by ERK1/2 protein association with PRC-occupied chromatin in mES cells [82], although this appears not to be conserved in human ES cells [84].

Here, we investigate the role of ERK1/2 signalling in the engagement of a spinal cord neural differentiation programme in the caudal mouse embryo and in human spinal cord neural progenitors generated in vitro. We find inhibition of ERK1/2 activity rapidly increases chromatin accessibility across an exemplar neural progenitor gene, *Pax6*, and establish that this gene is a polycomb target in vivo. We confirm that occupancy of PRC proteins at the *PAX6* locus also takes place in human NMP-like cells and show that ERK1/2 inhibition promotes precocious *PAX6* transcription. The transition to a *PAX6*-positive state coincides with loss of PRC occupancy, while ERK2 association with chromatin remains unchanged. Moreover, genome-wide analysis of chromatin accessibility (ATAC-seq) and polycomb occupancy (ChIP-seq for Ring1B) revealed that the decline in ERK1/2 activity promotes increased chromatin accessibility across thousands of neural genes, including a subset targeted by Ring1B in NMPs. Using genomic footprinting [85], we identify the most likely candidate factors engaged in the genome-wide response to ERK1/2 signalling decline, uncovering the succession of genome-wide, molecular steps that operate downstream of ERK1/2 signalling decline to facilitate and coordinate the onset of neural gene expression.

## Results

### Chromatin compaction around neural differentiation gene *Pax6* in mouse embryos is ERK1/2 dependent

To determine whether ERK1/2 signalling regulates chromatin organisation at neural differentiation gene loci in vivo, we exposed whole mouse embryos (E8.5) to the small molecule MEK inhibitor (MEKi) PD184352 or vehicle-only DMSO control for one hour (Fig 1B). The efficiency of ERK1/2 inhibition was determined by quantifying levels of phosphorylated ERK1/2 (42 and 44 kDa) in whole protein extracts from MEKi- and DMSO-treated embryos (Fig 1C and 1C’ and S1 Data). This regime was then used to measure chromatin compaction around the *Pax6* locus using FISH with fosmid probes hybridising either side of the locus [37] (Fig 1D). Chromatin compaction was assessed by measuring interprobe distance in *Pax6* transcribing neural tube cells and in CLE cells where *Pax6* has yet to be expressed (Fig 1D’ and 1D” and S1 Data). In untreated embryos, the interprobe distance was greater in neural tube than in CLE nuclei (Fig 1D’ and 1D” and S1 Data). Similar observations were made in the DMSO-treated embryos (Fig 1D’ and 1D” and S1 Data) and these two control conditions were not significantly different, suggesting that DMSO exposure did not alter chromatin organisation. By contrast, the *Pax6* locus was more open in CLE nuclei of MEKi-treated embryos compared to both controls (Fig 1D’ and 1D” and S1 Data). This decompaction correlated with a 1.7-fold decrease in the number of base pairs per nm compared to controls. These findings indicate that ERK1/2 activity promotes chromatin compaction at this neural differentiation gene locus and that active ERK1/2 is acutely required for this action in the embryo.

### Polycomb-mediated histone modification H3K27me3 marks the neural differentiation gene *Pax6* in vivo

To determine whether PRCs regulate chromatin compaction around the *Pax6* locus in vivo, we carried out chromatin immunoprecipitation (ChIP) for the PRC-mediated histone modification H3K27me3. Mouse embryo microdissection was performed to enrich for the caudal region at E8.5 for ChIP-qPCR (Fig 1E and see Methods). The *Pax6* transcription start site (TSS) was marked by high levels of H3K27me3 indicative of polycomb activity and consistent with repression of this gene in the CLE (Fig 1E’ and S1 Data). Similar levels were also detected at the known PRC target *HoxD11* [86–88], which is also not expressed in the mouse CLE at E8.5 [89]. By contrast, known PRC targets transcribed in the CLE, *HoxA7*, and *HoxB1* [90] exhibited minimal levels of H3K27me3 at their TSSs. This was similar to levels detected at a control intergenic region and the house-keeping gene *GAPDH* indicative of a lack of polycomb activity at these loci (Fig 1E’ and S1 Data). Together, these data demonstrate that polycomb activity at target genes is selective in vivo and identify *Pax6* as a polycomb target in caudal tissue. This raises the question of whether ERK1/2 activity directs polycomb occupancy and chromatin compaction and thus determines the onset of neural gene transcription.

### *PAX6* locus decompaction correlates with polycomb protein dissociation, H3K27me3 loss, and transcription onset in an in vitro model of human spinal cord differentiation

**Fig 2:**
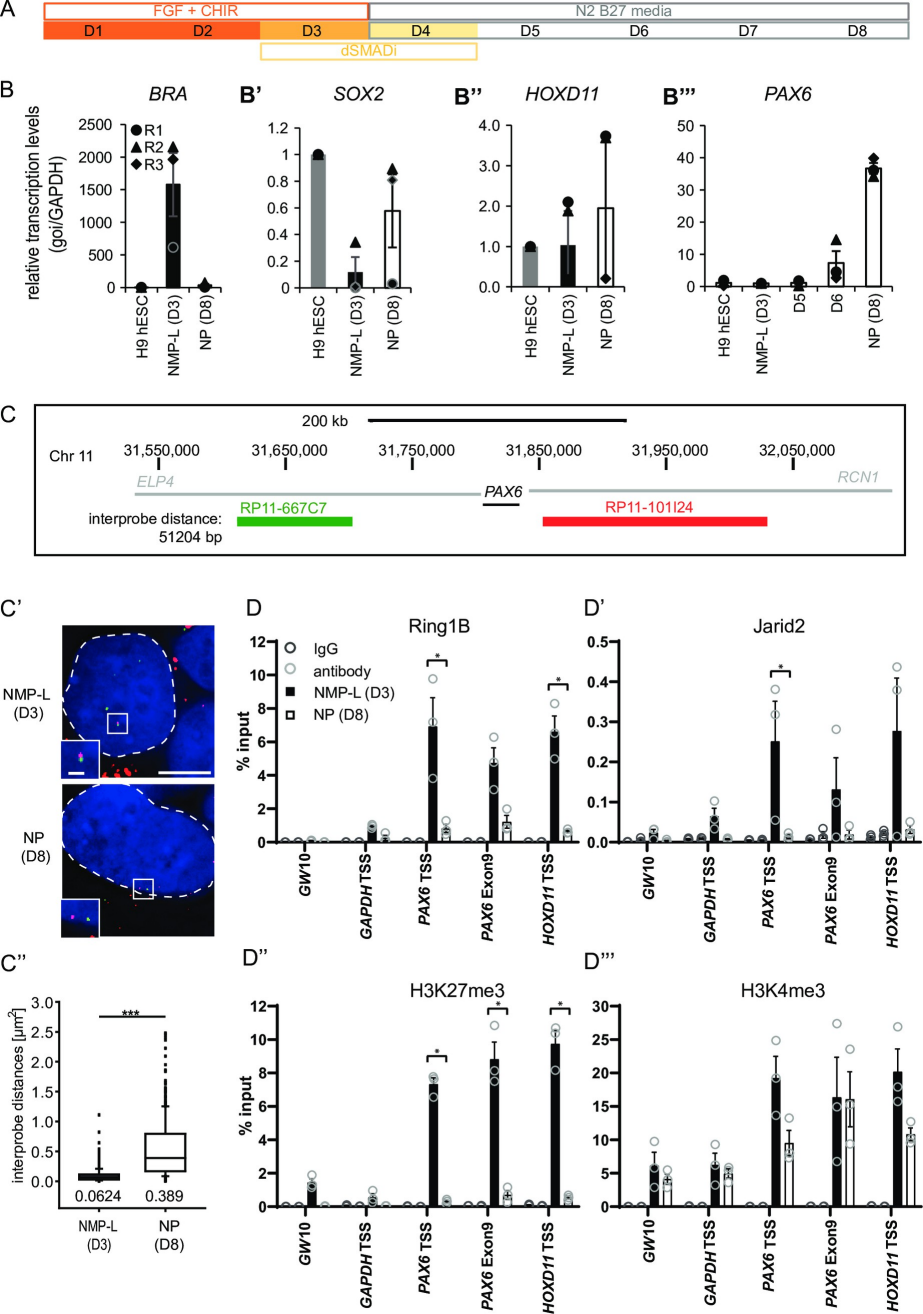
In vitro differentiation of human ESC-derived NMP-L cells to NPs reveals locus decompaction correlates with dissociation of polycomb repressive complexes and transcriptional onset at *PAX6*. (**A**) Schematic of differentiation regime used to generate first NMP-like cells (NMP-L (D3)) and then spinal cord progenitors (NPs (D8)) from human ESCs (D1 = Day 1. (**B**-**B”‘**) Transcription levels of *BRA*, *SOX2*, *HOXD11*, and *PAX6* assessed by RT-qPCR in undifferentiated cells (hESCs), NMP-Ls (D3), and NPs (D8) for *PAX6* additionally on day 5 (D5) and day 6 (D6) of differentiation (*n* = 3 independent experiments, indicated with circles, triangles, and diamonds, error bars = SEM) (S2 Data). (**C**-**C”**) FISH to assess chromatin compaction around the *PAX6* locus in NMP-L (D3) and NP (D8). Two probes flanking the target locus (interprobe distance ca. 51 kb) were hybridised and labelling visualised in DAPI-stained nuclei (blue, outlined with white dashed line). Interprobe distance measurements in >50 nuclei in NMP-Ls and NPs in 3 individual experiments (*n* = > 150 nuclei/cell type, Mann–Whitney test/rank-sum test, *** *p* ≤ 0.001) (S2 Data). (**D**-**D”‘**) ChIP-qPCRs investigating polycomb repressive complex occupancy (Ring1B/PRC1 and Jarid2/PRC2) and the histone modifications H3K27me3 and H3K4me3 in NMP-L and NP cells (note, D8 Jarid2 is below IgG control) (*n* = 3 independent experiments, each indicated by circles), bar = average, error bar = SEM, * *p* ≤ 0.01, *t* test (S2 Data). ChIP-qPCR, chromatin immunoprecipitation quantitative PCR; ESC, embryonic stem cell; FISH, fluorescent in situ hybridisation; IgG, immunoglobulin G; NMP-L, NMP-like; NP, neural progenitor; RT-qPCR, reverse transcription quantitative PCR.

To test this, we developed an in vitro system to study neural differentiation during the generation of the spinal cord. We reasoned that the use of human ESCs, with the slower progression to neural progenitor cell identity, would provide a better temporal resolution of mechanisms mediating neural differentiation. To this end, we directed the differentiation of human ESCs towards spinal cord using a protocol established previously [91,92] (Fig 2A and see Methods). This involved the generation of NMP-like cells on day 3 (NMP-L D3) (characterised by the coexpression of BRA and SOX2, which are analogous to NMP cells in the mouse CLE) and their differentiation into *PAX6* expressing neural progenitors by day 8 (NP D8), which also transcribed the known PRC target *HOXD11* (Fig 2B–2B” and S2 Data). Detailed analysis of this differentiation revealed low level onset of *PAX6* on D6, anticipating the robust expression detected on D8 (Fig 2B”’). This neural differentiation was further slowed by not including exogenous retinoic acid, which acts in part by inhibiting FGF/ERK signalling; this modification allowed us to test the ability of MEK inhibition/loss of ERK1/2 activity to promote neural differentiation (Fig 2A and see Methods). Having established this assay, we next asked whether changes in chromatin compaction that we observe around the *Pax6* locus in mouse embryo also occur at *PAX6* during human neural differentiation in vitro (Fig 1D). FISH was carried out in NMP-L (D3) and NPs (D8) cells, and the distances between the *PAX6*-flanking probes were measured (Fig 2C–2C” and S2 Data). This revealed a significant increase in interprobe distance in NPs (D8) compared to NMP-Ls (D3) (Fig 2C’ and 2C” and S2 Data). This indicates that the regulation of chromatin compaction is a conserved mechanism operating during neural differentiation that can be recapitulated and examined in vitro.

To elucidate how chromatin compaction is regulated at the *PAX6* locus, we interrogated PRC occupancy over developmental time using ChIP-qPCR in NMP-L (D3) cells versus NPs (D8). We assessed the occupancy of the core PRC1 protein Ring1B (which is required in the embryonic mouse epiblast that contains NMPs, while Ring1A loss affects only later development) [74] and for PRC2, Jarid2 (as the latter is highly expressed in the NMP containing caudal lateral epiblast and newly forming neural progenitors in chick [93] and mouse embryos (S1 Fig)). Together with H3K27me3, this analysis established that PRC proteins are present at the TSS and along the gene body of *PAX6* and at the TSS of known PRC target *HOXD11* in NMP-L (D3) cells. By contrast, reduced occupancy was detected at *PAX6* and *HOXD11* in NPs (D8) (while baseline detection in the gene desert region (Gw10) and the housekeeping gene *GAPDH* remained unchanged) (Fig 2D-D” and S2 Data). Strikingly, analysis of the active histone modification H3K4me3 revealed no change at the *HOXD11* or *PAX6* TSSs during neural differentiation (Fig 2D” and S2 Data). This suggests that polycomb-mediated repression is a major activity restricting *PAX6* gene expression in the NMP-L cells, and not the low levels of H3K4me3. Together, these findings demonstrate that site-specific deposition of PRC at *PAX6* is transient and that its removal correlates with chromatin decompaction at this locus, characteristic of the neural progenitor cell state.

### Progressive global increase in neural gene accessibility during in vitro human differentiation

**Fig 3:**
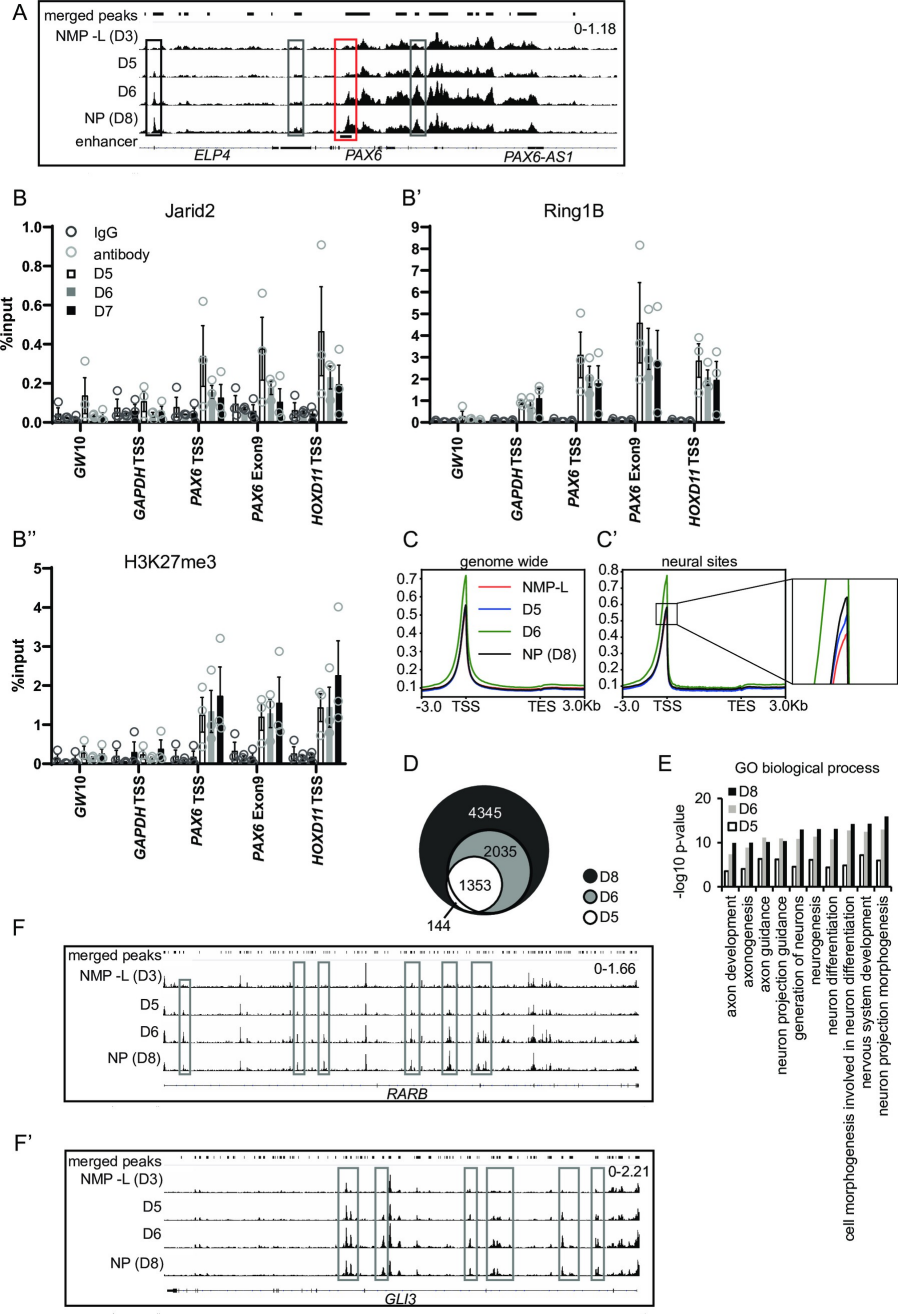
Progressive increase in human neural gene accessibility during in vitro differentiation. (**A**) ATAC-seq peak tracks in NMP-L (D3), D5, D6, and NP (D8) cells for the genomic regions of *PAX6*, grey boxes are peaks within the gene body, black boxes, peaks outside of the gene body and red box genomic region of a known *PAX6* enhancer. (**B**-**B”**) ChIP-qPCR for Jarid2, Ring1B, and H3K27me3 on D5, D6, and D7, note by D7 Jarid2 signal falls below detection (*n* = 3 independent experiments, each indicated by circles), bar = average, error bars = SEM, although there is a declining trend for Jarid2, there are no significant differences between samples, *t* test (S3 Data). (**C**-**C’**) Metaprofiles comparing chromatin accessibility in NMP-L (D3) and NP (D8) cells along the gene body genome-wide (C) and focussed on genes associated with increased accessibility in NPs (D8) (neural sites, C’) compared to NMP-L (S3 Data). (**D**) Venn diagram representing distribution of genomic regions more accessible in NPs (D8) (neural sites) compared to NMP-L (D3) and their appearance over time (D5 and D6). Comparison of accessible regions and associated genes on D5 and after D5 revealed approximately 19% of genes had additional accessible regions, while the majority were found in newly accessible genes, similarly 40% of D6 genes with additional regions became more accessible in NP (D8). (**E**) GO term analysis of genes associated with neural sites compared to NMP-L (D3) and genes associated with more accessible chromatin regions on D5 and D6 out of the neural sites (S3 Data). (**F**-**F’**) ATAC-seq peak tracks in NMP-L (D3), D5, D6, and NP (D8) cells for the genomic regions of RARB (E) and GLI3 (E’), grey boxes are peaks within the gene body. ChIP-qPCR, chromatin immunoprecipitation quantitative PCR; GO, gene ontology; NMP-L, NMP-like; NP, neural progenitor.

While these data indicate that PRC loss precedes neural gene transcription, it remained unclear whether the regulation of chromatin accessibility is specific for *PAX6* or reflects a general mechanism that mediates onset of neural gene transcription. To address this, we performed a global analysis of the chromatin accessibility landscape using ATAC-seq [94], as cells differentiated into NPs (D8) from the NMP-L (D3) state. This involved differentiation of NMP-L (D3) cells (as in Fig 2A) and sampling cell populations at intervals (D5, D6, and D8). Analysis of the chromatin configuration across the *PAX6* locus revealed increased accessibility at multiple sites, some as early as D5 and most prominent by D6 (Fig 3A). These early increases in chromatin accessibility prefigure reduction in PRC protein occupancy at the *PAX6* locus detected by ChIP-qPCR, which only becomes significant by D8 (Fig 3B and 3B’ and S3 Data; and also see Fig 2D and 2D’). In contrast to the declining trend in levels of Jarid2 occupancy during differentiation, H3K27me3 levels at the *PAX6* TSS and along the gene body remained unchanged between D5 and D7 (Fig 3B’), only dropping later at D8 (Fig 2D”). Thus, a reducing trend in PRC occupancy, likely reflecting differentiation progression of some cells, is observed concurrent with increased chromatin accessibility, while loss of H3K27me3 appears to be a later step that coincides with robust *PAX6* transcription.

Genome-wide comparison of accessibility patterns across TSSs and gene bodies in D5, D6, and D8 cells revealed that although overall accessibility levels remained relatively constant over time, a clear peak in accessibility appeared at D6 of differentiation (Fig 3C). Detailed analysis comparing D3 and D8 identified 7,877 regions with increased, and 11,603 regions with decreased accessibility (analysis performed with diffReps with thresholds FDR ≤0.01 and log2FC ≥1). The regions of increased accessibility correlated with known active enhancer and promoter marks such as H3K4me1 and H3K27ac detected in human embryonic spinal cord and brain (data from the ENCODE regulatory element database [95]; see S2 Fig and S4 Data). We termed these regions “neural sites” and further analysis revealed that these were located in introns (7,003), exons (333), promoter/TSSs (199), TTS (175), and intergenic regions (167), while additional comparison with ENCODE datasets for human neurons and hESCs identified overlaps between neural sites and peaks for the transcriptional coactivator p300, indicating that recruitment of this histone acetyltransferase is a potential mechanism for these changes in chromatin accessibility. Overall, we found that neural sites were associated with 4,001 genes (S1 Table) and that accessibility increased across the TSS and body of these genes over time, with accessibility peaking at D6 (Fig 3C’ and S3 Data). The changes included additional chromatin opening across genes already accessible at D3 as well as new genes with increased accessibility by D8 (Fig 3D).

Gene ontology (GO) term analysis of the genes associated with regions of increased accessibility on D5, D6, and D8 revealed terms related to neural development and showed a progression with more genes associated with these terms over time (Fig 3E and S3 Data, gene list S2 Table). Focussing on the GO term Neurogenesis, we selected 2 key neural progenitor genes, in addition to *PAX6*, and examined the ATAC-seq signal in detail around their gene bodies. Chromatin accessibility around *RARB* and *GLI3* showed an increase similar to that associated with *PAX6* as early as D5 (Fig 3F and 3F’). Overall, these findings indicate that coordinated regulation of chromatin accessibility is a general mechanism that mediates the onset of neural gene transcription, while analysis of PRC occupancy dynamics at the exemplar *PAX6* locus identifies this repression complex as a target of such regulation.

### ERK1/2 dephosphorylation induces precocious neural gene transcription and polycomb protein dissociation

**Fig 4:**
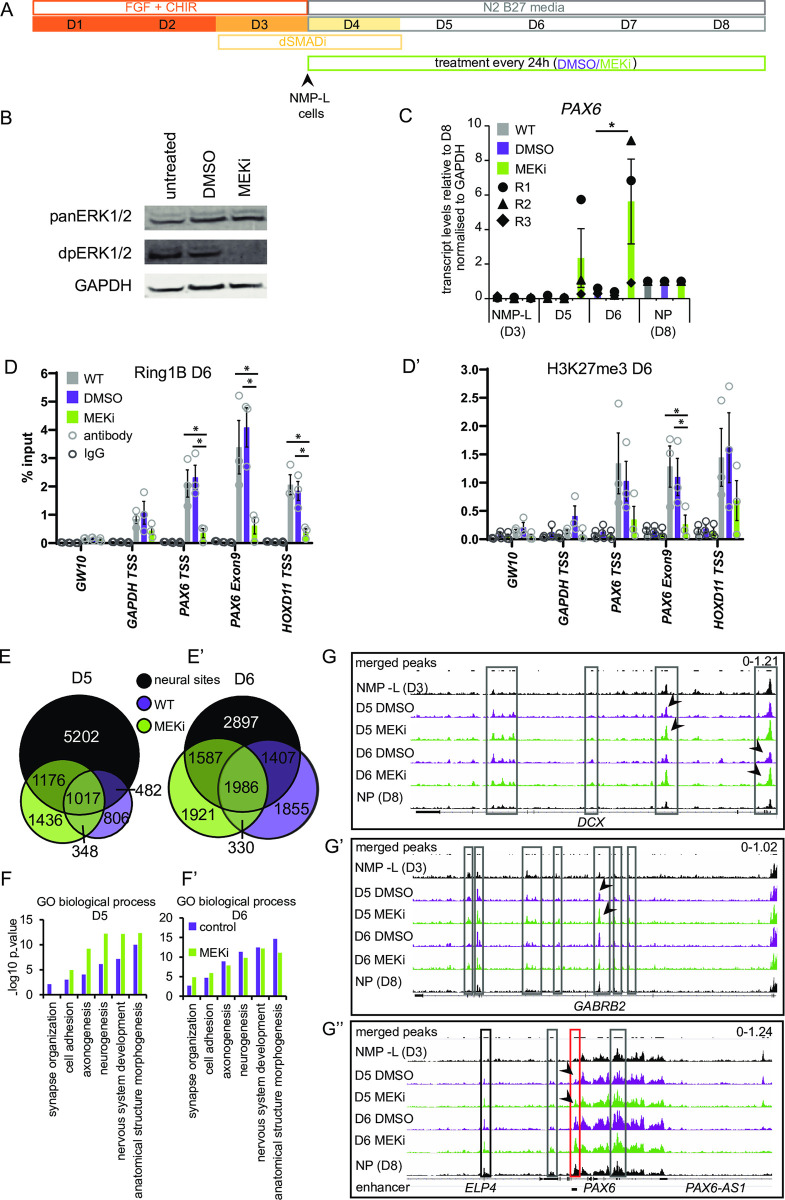
ERK1/2 dephosphorylation induces precocious *PAX6* expression, reduced polycomb protein occupancy and H3K27me3 at this locus and increases accessibility of hundreds of neural differentiation genes. (**A**) Differentiation protocol generating NMP-L (D3) cells and spinal cord NPs by D8 and additional treatment with vehicle control DMSO or MEKi (PD184352) every 24 hours from D3 for the duration of the differentiation protocol. (**B**) Western blot confirming that exposure to MEKi lead to reduced ERK1/2 phosphorylation after 24 hours in differentiation conditions (D3-D4). (**C**) *PAX6* transcript levels in NMP-L (D3), in control conditions (WT and DMSO) or in MEKi-treated cells on D5 and D6 and NP(D8) determined by RT-qPCR (*n* = 3 independent experiments indicated by circles, triangles, and diamonds, bar = average, error bars = SEM) (S5 Data). (**D**-**D’**) ChIP-qPCR for Ring1B and H3K27me3 on D6 note H3K27me3 below detection in MEKi conditions (*n* = 3 independent experiments indicated with circles, bar = average, error bars = SEM, * = *p* ≤ 0.05, *t* test, S5 Data). (**E**-**E’**) Venn diagrams representing distribution of genomic regions that become more accessible on D5 (E) and D6 (E’) in control condition and MEKi-treated cells compared to NMP-L (not restricted to regions accessible in NPs (D8)) as well as more accessible regions on D8. (**F**-**F’**) GO term analysis of genes associated with more accessible chromatin in D5 (F) and D6 (F’) in control condition and MEKi-treated cells compared to NMP-L (S5 Data). (**G**-**G”**) ATAC-seq peak tracks in NMP-L, D5 DMSO and MEKi, D6 DMSO and MEKi, and NP (D8) cells for the genomic regions of *DCX* (G), *GABRB2* (G’), and *PAX6* (G”), grey boxes outline peaks within the gene body, black boxes peaks outside of the gene body and red box genomic region of a known *PAX6* enhancer, example comparisons between peaks in MEKi and DMSO conditions indicated with black arrowhead pairs. Note MEKi exposure does not alter levels of Ring1B protein while it promotes neural differentiation (S4 Fig). ChIP-qPCR, chromatin immunoprecipitation quantitative PCR; GO, gene ontology; MEKi, MEK inhibitor; NMP-L, NMP-like; NP, neural progenitor; RT-qPCR, reverse transcription quantitative PCR; WT, wild type.

In chick and mouse embryos, FGFR inhibition elicits precocious *Pax6* transcription [20,37], and loss of such signalling [37] or downstream ERK1/2 activity (this study) also decompacts this gene locus in mouse embryo caudal lateral epiblast. We therefore next tested whether loss of ERK1/2 signalling similarly accelerates human neural differentiation from NMP-L (D3) cells in vitro and if such signalling regulates polycomb protein occupancy. To address this, NMP-L (D3) cells were generated and exposed to MEKi or DMSO during subsequent neural differentiation (Fig 4A and 4B) and *PAX6* transcript levels analysed by reverse transcription quantitative PCR (RT-qPCR). This revealed precocious *PAX6* transcription beginning at D5 and peaking at D6 in the presence of MEKi (Fig 4C and S5 Data). This correlated with changes in polycomb protein occupancy and activity by D6, where MEKi exposure resulted in decreased Ring1B and H3K27me3 at the *PAX6* and control locus *HOXD11* (Fig 4D and 4D’ and S5 Data) (while ChIP-PCR for Jarid2 provided low signal at D6, which may reflect decreased levels of Jarid2 protein in NPs; S3A and S3B Fig and S6 Data). Importantly, these changes coincide with maximum precocious *PAX6* transcription elicited by inhibition of ERK1/2 signalling (Fig 4C and S5 Data).

### ERK1/2 dephosphorylation impacts polycomb occupancy without altering global Ring1B levels or ERK2 association with chromatin

These findings identify ERK1/2 signalling as a regulator of PRC occupancy and transcription of a key neural gene. This might involve mechanisms that influence the activity of PRC proteins but could be mediated by regulation of PRC protein expression. As Ring1B occupancy is significantly reduced by D6 (Fig 4D’ and S5 Data), we assessed levels of Ring1B by western blot during differentiation from hESCs. This revealed a sharp decline as hESCs become NMP-L cells and a further small decrease in Ring1B as NMP-L (D3) cells become NPs (D8) (S4A and S4B Fig and S7 Data). This raised the possibility that reduction in global levels of Ring1B contribute to reduced PRC occupancy and that loss of ERK signalling might accelerate differentiation by reducing Ring1B expression. To test whether ERK signalling regulates Ring1B levels, we repeated our initial experiment treating cells with MEKi for 3 days (D3-D6 as in Fig 4A). This regime reduced pERK1/2 and induced precocious *PAX6* expression (S4C and S4D Fig and for comparison, also see Fig 4C and S7 Data) but did not alter Ring1B protein levels. This indicates that ERK signalling decline does not impact PRC occupancy by regulating expression or turnover of Ring1B.

A more direct mechanism by which ERK1/2 signalling might regulate PRC occupancy has been proposed in mouse ESCs. Here, ChIP-seq experiments revealed that ERK1/2 activity promotes ERK2 occupancy at developmental genes, in addition to the recruitment of polycomb proteins at such genes in an ERK2-dependent manner [82]. Using ChIP-qPCR and the same ERK2 antibody used and validated in [82], we surveyed ERK2 occupancy during differentiation from NMP-L (D3) cells. ERK2 was detected at TSSs of *PAX6*, *HOXD11* and the constitutively expressed control gene *GAPDH* (S6A Fig). Although ERK2 occupancy at *PAX6* and *HOXD11* declined during differentiation (S4G Fig and S7 Data), inhibition of ERK1/2 activity revealed no difference in ERK2 occupancy between treated and control samples (S4H and S4H’ Fig and S7 Data). These findings suggest that a decline in ERK1/2 activity, and not its association with chromatin, regulates PRC occupancy at the exemplar neural gene *PAX6* in this cellular context.

### ERK1/2 dephosphorylation induces global increase in chromatin accessibility at neural sites and predicted changes in occupancy of transcription factors that mediate transition to a neural identity

To determine whether changes to ERK1/2 activity broadly impact chromatin accessibility in cells transitioning to a neural identity, we performed ATAC-seq on cells at D5 and D6 exposed to MEKi or DMSO conditions during differentiation from D3 NMP-L cells (Fig 4A). A comparison of all regions opening between NMP-L (D3) and D5 in control (2,653 regions) and MEKi conditions (3,977 regions, associated with 417 newly accessible genes, and 368 more accessible genes), identified 2,193 regions with additional chromatin accessibility following MEKi treatment (Fig 4E and S3 Table). Many of the MEKi-induced regions of increased accessibility belonged to the previously defined neural sites (1,176) (Fig 3C’), while a further (1,436) regions were unique to MEKi treatment and not included in neural sites (Fig 4E). Analysis of D6 control and MEKi datasets revealed a similar pattern, with 3,508 additional regions of increased accessibility induced by MEKi (associated with 593 newly accessible genes and 437 genes acquiring additional accessibility) (Fig 4E’), with many coinciding with neural sites (1,587), and a further 1,921 regions were detected outside of control D6 and D8 data sets (Fig 4E’ and see below).

To interpret the genome-wide chromatin accessibility changes induced by MEKi, we analysed the GO biological processes associated with the gene cohorts linked to regions of increased accessibility. Strikingly, MEKi treatment on D5 corresponded to an increased representation of genes associated with neural development terms (Fig 4F and S5 Data). Moreover, at D6 of MEKi treatment, genes associated with later neural development, such as synapse organisation, were specifically increased (Fig 4F and S5 Data). We therefore analysed the GO terms for genes associated with more accessible regions that were unique to the MEKi data sets on D5 and D6 and found a strong enrichment towards later neuronal differentiation (see S3 Table for list of genes associated with loci of increased accessibility due to MEKi treatment). This suggests that MEKi treatment leads to increased global accessibility at neural differentiation genes and an accelerated progression to a more advanced differentiation cell state, consistent with MEKi-induced precocious *PAX6* transcription and loss of Ring1B across this locus shown by ChIP-PCR by D6 (Fig 4C, 4D, and 4D’ and S5 Data). Moreover, examining the peaks detected by ATAC-seq at individual genes (*DCX*, *GABRB2*, and *PAX6*) supported these conclusions, showing an increase over time and that this was advanced with MEKi treatment assessed on D5 (arrowheads in Fig 4G–4G”).

**Fig 5:**
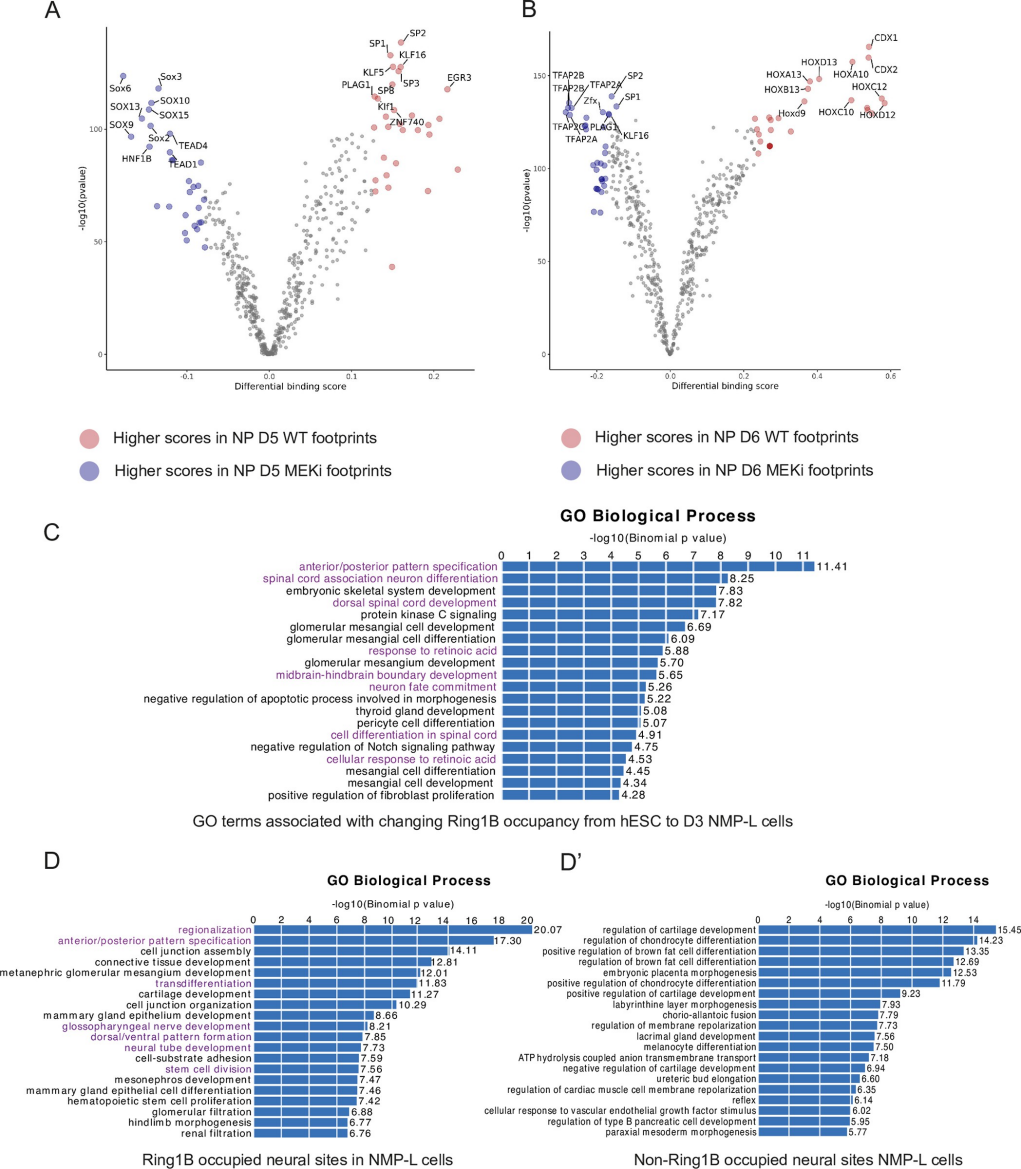
Bioinformatic analyses predict engagement of SOX factors following MEKi exposure and reveal distinct gene pathways occupied by RING1B in NMPs. Top ten TFs identified by genomic footprinting of regions of increased accessibility detected by ATAC-seq following exposure to MEKi or control (WT) conditions on (**A**) D5 and (**B**) D6 (S8 Data); GO biological processes detected by GREAT; (**C**) that are associated with genomic intervals that change in Ring1B occupancy as detected by ChIP-seq between day 0 hESCs and day 3 NMPs-L cells (see Method); and associated with neural sites occupied by (**D**) Ring1B (purple—enriched for neural and patterning genes) and (**D’**) non-Ring1B in NMP-L cells. GO, gene ontology; hESC, human ESC; MEKi, MEK inhibitor; NMP, neuromesodermal progenitor; NMP-L, NMP-like; NP, neural progenitor; TF, transcription factor; WT, wild type.

To assess how MEKi treatment might advance neural differentiation, we next analysed its impact on the repertoire of transcription factors (TFs) found at open chromatin sites. For this, we carried out genomic footprinting, a computational approach that predicts TF occupancy from ATAC-seq data on the basis of the Tn5 transposase integration frequency detected at base-pair resolution, combined with motif enrichment [85]. We assessed all accessible regions detected by ATAC-seq at D5 and D6, comparing control versus MEKi-treated conditions. This revealed an enrichment in neural TFs in MEKi conditions, notably SOX factors [96] at D5, as well as TFAP2A by D6, indicative of a neural crest-like signature [97]. By contrast CDX/HOX factors, associated with the NMP-L state [98], were found in control conditions (Fig 5A and 5B and S8 Data). These data suggest that MEKi exposure promotes global changes in the binding of TFs that mediate the transition to a neural identity. Moreover, a MEKi-induced increase in SOX factor binding at D5 occurs prior to precocious *PAX6* expression on D6, suggesting that neural TF binding is an earlier distinct step that precedes neural gene transcription.

### Changing patterns of Ring1B occupancy correlate with establishment of the NMP-L cell state and show that ERK1/2 decline regulates PRC and other mechanisms to increase chromatin accessibility at neural genes

To uncover the global relationship between PRC-occupied genes and subsequent sites of increased accessibility, we next undertook ChIP-seq with Ring1B in hESCs and D3 NMP-L cells. Comparison of Ring1B genome-wide occupancy in these two cell states revealed a striking pattern of change, with 1,827 sites detected as differential. Most of these sites (1,148) were decreased in NMP-L cells in comparison with hESCs, with a smaller set (679) constituting newly occupied sites in NMP-L cells. Such change in occupancy pattern further supports the conclusion that reduced Ring1B at the *PAX6* locus (Fig 4D and S5 Data) is not simply a consequence of overall decline in Ring1B protein levels during differentiation. Moreover, GO analysis of genes associated with these differentially Ring1B occupied sites included many terms associated with spinal cord development and neural differentiation (Fig 5C). This establishment of a new pattern of genome wide Ring1B occupancy in the NMP-L (D3) cells that is aligned with spinal cord development supports the idea that the NMP cell state represents competency for differentiation into this tissue and suggests that this is linked to regulation of polycomb occupancy.

To assess the significance of Ring1B occupancy in NMP-L (D3) cells, we next compared all such sites (D3 consensus sites) to our previously (ATAC-seq defined) neural sites, which exhibit increased accessibility from D3 to D8. This revealed that a subset of neural sites (22%, 1,698/7,877) are occupied by Ring1B in the NMP-L cell population, while GO term analysis revealed enrichment for neural and anterior-posterior patterning associated with these Ring1B bound sites (Fig 5D and 5D’), linking PRC occupancy in NMP-L (D3) cells to neural differentiation. On the other hand, Ring1B occupancy was not predictive of which neural sites would show increased accessibility following exposure to MEKi: Only a subset of neural sites opening in response to MEKi were bound by Ring1B on D5 (258/1,176, 15%, includes *PAX6*) and D6 (373/1,587, 21.9%, includes *PAX6* as well). This suggests that MEKi increases chromatin accessibility at canonical PRC and non-PRC-occupied sites.

To test whether Ring1B occupancy in NMP-L cells correlates with the binding of specific TFs, we further performed genomic footprinting on the Ring1B-bound subset and compared this with all other neural sites but found no differences in the factors enriched. However, comparison of predicted TF occupancy in MEKi and control conditions revealed that loss of ERK1/2 activity promoted the engagement of SOX family TFs (D5) and TFAP2 family TFs (D6) regardless of association with Ring1B. These findings indicate that while a decline in ERK1/2 signalling promotes increased accessibility at neural gene loci and promotes engagement of neural TFs, only a subset of the newly accessible loci was formerly occupied by Ring1B. Moreover, these data suggest that the activity of individual TF proteins such as SOX2 and SOX3 is subject to multiple regulatory mechanisms at different genomic sites. Overall, these data show that decline in ERK signalling broadly impacts accessibility at neural genes, can regulate PRC occupancy, and selectively promotes neural TF engagement signatures at neural gene loci.

### Transient ERK1/2 dephosphorylation in NMP-L cells induces dissociation of both Jarid2 and Ring1B and chromatin decompaction at the *PAX6* locus but does not alter H3K27me3 nor elicit transcription

In the mouse embryo, we found that exposure to MEKi led to decompaction of chromatin around the locus of our exemplar neural progenitor gene *Pax6*, within one hour (Fig 1). However, it was not practical to dissect and collect sufficient caudal embryonic tissue to assess the impact of loss of ERK1/2 signalling on PRC protein occupancy or the timing of such events. We therefore used human NMP-L cells to dissect the dynamics of such changes at the *PAX6* locus. Exposure to MEKi for 3 hours (in the presence of FGF, which maintains the NMP-L cell state) reduced dpERK1/2 levels (S5A–S5B’ Fig and S9 Data); however, ChIP-qPCR revealed no change in Ring1B nor H3K27me3 occupancy (S5C–S5E and S9 Data). Moreover, MEKi exposure did not alter transcription of *PAX6*, *HOXD11*, nor *JARID2* (S5F-S5F” Fig and S9 Data). This short exposure was therefore insufficient to disassemble the PRC1 (Ring1B) complex or remove the H3K27me3 mark in the context of NMP-L (D3) cells.

**Fig 6:**
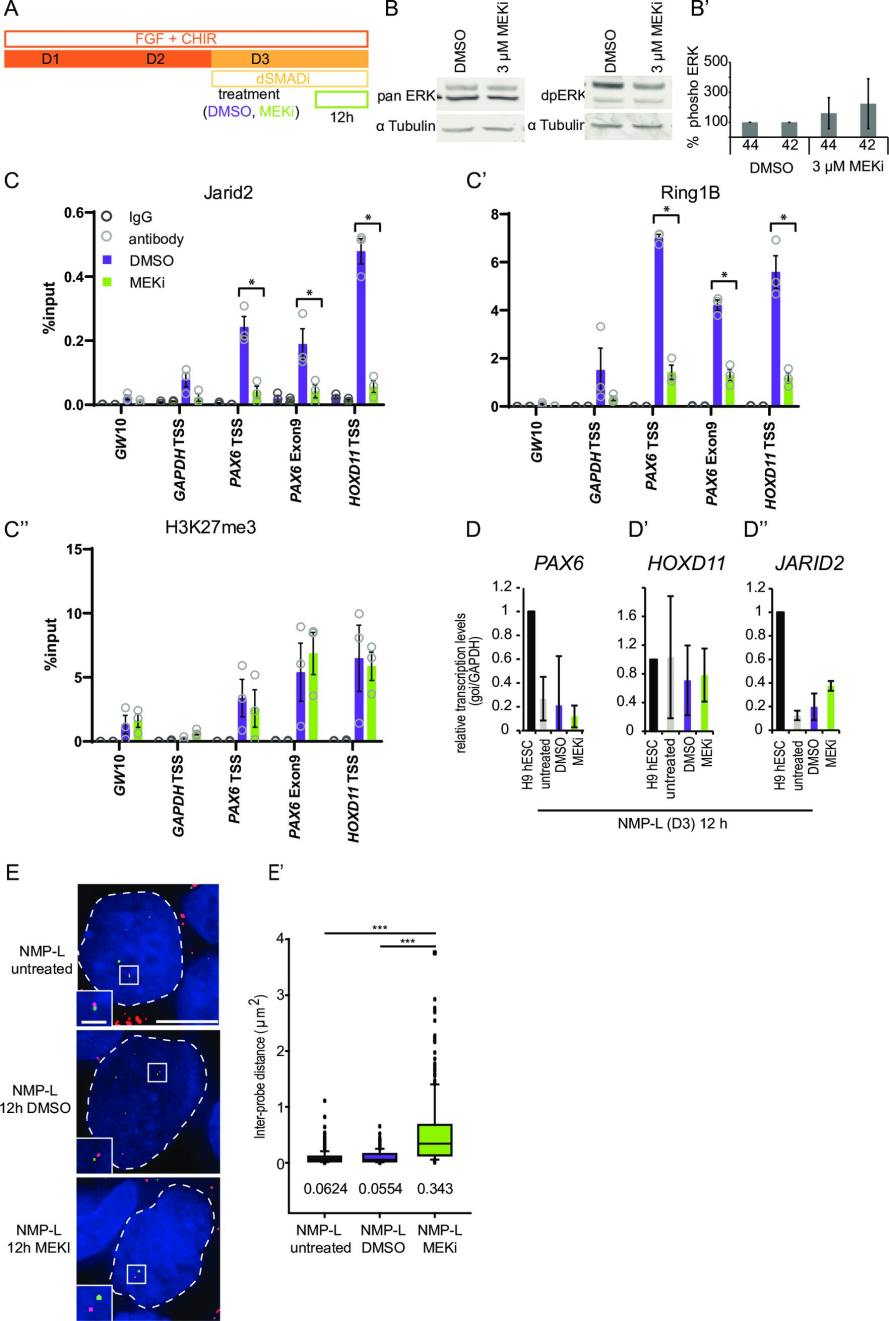
Transient ERK1/2 dephosphorylation induces dissociation of Jarid2 and Ring1B and chromatin decompaction in NMP-L cells at the *PAX6* locus but does not reduce H3K27me3 nor elicit transcription. (**A**) Differentiation protocol used to generate NMP-L (D3) cells and additional treatment with vehicle control DMSO or MEKi for 12 hours. (**B**-**B’**) Representative western blot of cell lysates probed with antibodies against total (panERK1/2) and dual-phosphorylated-ERK1/2 (dpERK1/2) and LiCOR quantification data (n = 3 independent experiments, error bar = SEM) (S10 Data). (**C**-**C”**) ChIP-qPCRs investigating the histone modification H3K27me3 and polycomb repressive complex occupancy in NMP-L (D3) cells treated with MEKi or DMSO for 12 hours (*n* = 3 individual experiments indicated with circles, bar = average, * = *p* ≤ 0.05, *t* test) (S10 Data). (**D**-**D”**) Transcription levels of *PAX6*, *HOXD11*, and *JARID2* assessed by RT-qPCR in undifferentiated cell (hESCs), NMP-L cells untreated, vehicle control (DMSO) treated or MEKi treated (*n* = 3 individual experiments, no significant differences between samples, *t* test) (S10 Data). (**E**-**E’**) FISH to assess chromatin compaction around the *PAX6* locus in NMP-Ls untreated and treated with DMSO or MEKi for 12 hours. Two probes flanking the target locus (interprobe distance ca. 51 kb) were hybridised and visualised by differential labelling nuclei (outlined with white dashed line) visualised with DAPI (blue). Interprobe distance measurements in >50 nuclei in the three conditions in three individual experiments (*n* = > 150 nuclei/cell type, Mann–Whitney test/rank-sum test, *** *p* ≤ 0.001) (S10 Data), this decompaction correlated with a 2.5-fold decrease in number of base pairs per nm compared to both controls (untreated NMP-L (D3): 205 bp/nm, DMSO NMP-L (D3): 218 bp/nm and MEKi NMPL (D3): 87 bp/nm). ChIP-qPCR, chromatin immunoprecipitation quantitative PCR; MEKi, MEK inhibitor; NMP-L, NMP-like; RT-qPCR, reverse transcription quantitative PCR.

We next assessed the consequences of loss of ERK1/2 activity at a later time point, exposing NMP-L (D3) cells to MEKi for 12 hours and conducting the same panel of ChIP-qPCR experiments (Fig 6A). At the end of this period, ERK1/2 phosphorylation levels had now returned to control levels (Fig 6B and 6B’ and S10 Data), indicative of transient MEKi effectiveness in the presence of exogenous FGF, although other consequences of reduced ERK1/2 activity, such as increased PKB phosphorylation, were still apparent (S6 Fig and S11 Data): This regime therefore delivered a transient inhibition of ERK1/2 activity for at least 3 hours. Moreover, after 12 hours, reduced Jarid2 and Ring1B occupancy were clearly apparent at *PAX6* and *HOXD11* loci in exposed cells (while control genomic regions possessed only low level H3K27me3 and Ring1B and Jarid2, which did not change with MEKi treatment) (Fig 6C–6C’ and S10 Data). Importantly, none of the gene loci analysed exhibited a change in H3K27me3 levels (Fig 6C” and S10 Data) nor did the loss of both PRC1 and PRC2 proteins correlate with transcription of *PAX6* or *HOXD11* nor with reduced *JARID2* transcripts (Fig 6D–6D” and S10 Data). These data indicate that a transient reduction in ERK1/2 signalling is sufficient to trigger loss of both PRC2 and PRC1 proteins.

**Fig 7:**
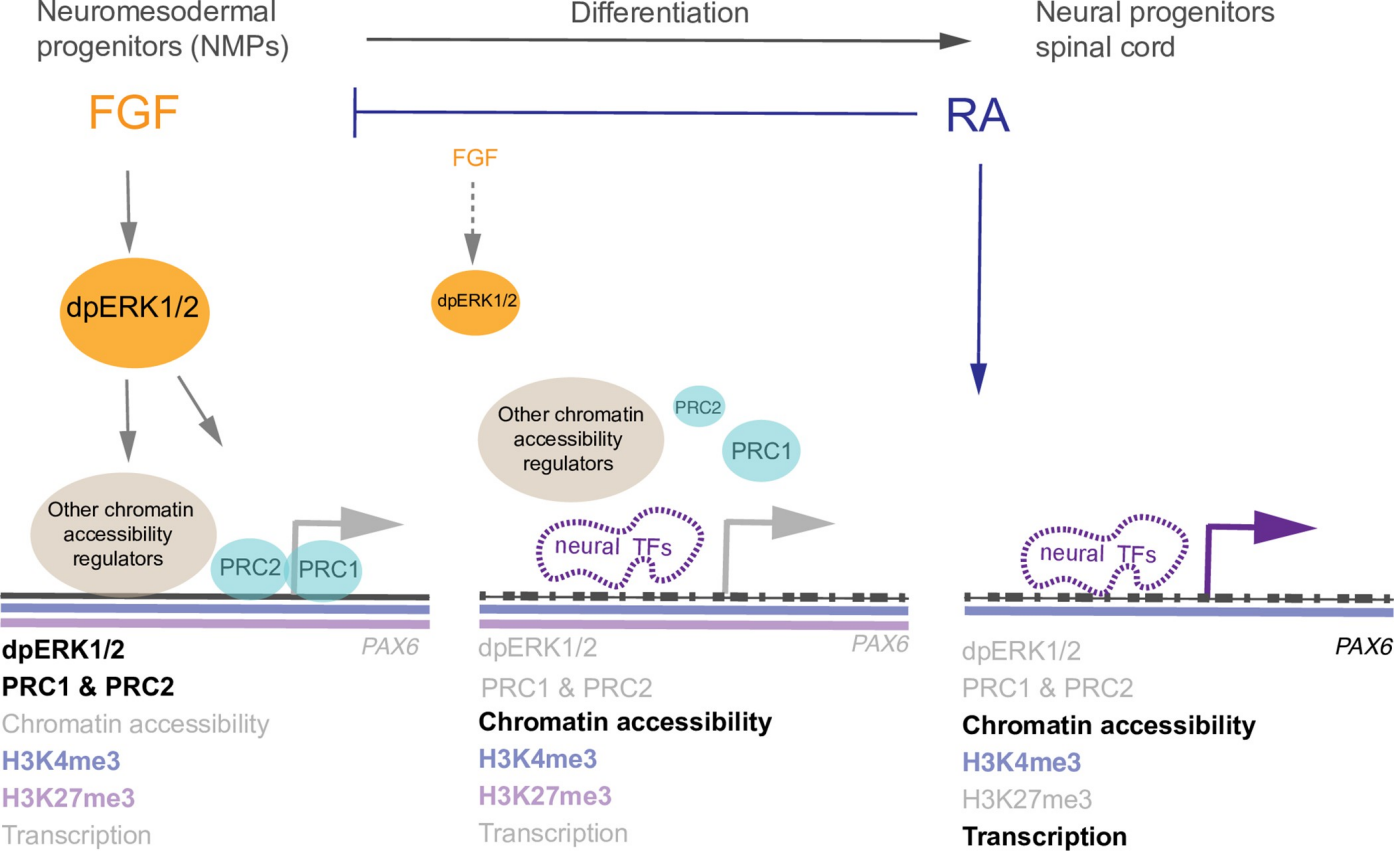
Model of steps leading to engagement of the neural differentiation programme at exemplar gene *PAX6*. In chick and mouse embryos, decline in FGF and downstream effector kinase ERK1/2 signalling takes place as NMP cells commence neural differentiation, mediated by rising levels of retinoid (RA) signalling, which represses *Fgf8* (reviewed in [25]). Mouse NMPs are characterised by phosphorylated ERK1/2 (dpERK1/2) and compact chromatin across the exemplar neural differentiation gene *Pax6*, which is decorated with the PRC2 gene silencing mark H3K27me3. In human NMP-L cells, PRC2 (Jarid2) and PRC1 (Ring1B) occupy the locus and H3K27me3 is accompanied by gene activation associated histone modification H3K4me3, identifying this as a bivalent locus poised for transcription. During differentiation, loss of ERK1/2 signalling leads to chromatin decompaction across *PAX6* in mouse embryo NMPs and in human NMP-L cells, where it is accompanied by loss of PRCs (PRC2 and PRC1). There is also evidence that levels of PRC2 protein Jarid2 decline during neural differentiation. Loss of ERK1/2 activity results in genome-wide increase in chromatin accessibility across neural genes, involving regulation of PRC and other chromatin accessibility complexes, opening sites that become bound by neural TFs (SOX family), detected prior to *PAX6* transcription. The gene silencing histone mark H3K27me3 persists after PRC2 is lost and is removed coincident with transcription onset: ERK1/2 regulation of chromatin accessibility and neural TF binding appears distinct from this later step, which may depend on retinoid signalling, known to be required for *PAX6* transcription in mouse and chick embryos. As transient loss of ERK1/2 activity removes PRC2 and PRC1 and these complexes are not reinstated on resumption of ERK1/2 signalling, decline in ERK1/2 activity and so PRC occupancy (and other chromatin remodelling complexes) acts as a gating mechanism that confers differentiation directionality as well as synchronising accessibility for neural TF binding and so engagement of the neural differentiation programme. FGF, fibroblast growth factor; NMP, neuromesodermal progenitor; NMP-L, NMP-like; RA, retinoic acid; PRC, polycomb repressive complex; TF, transcription factor.

To determine the significance of this loss, we assessed whether 12-hour MEKi exposure also altered chromatin accessibility around the *PAX6* locus using FISH. Significant decompaction of this region was found in MEKi-treated cells compared with both DMSO and untreated controls (Fig 6E–6E’ and S10 Data). These findings correlate PRC loss with a distinct increase in chromatin accessibility across this key neural progenitor gene. This loss of PRC occupancy appears to represent the gating mechanism for the initiation of neural progenitor gene expression since the resumption of ERK1/2 activity (by 12 hours) did not reinstate PRC binding. Moreover, these data demonstrate that removal of H3K27me3 is not required for such chromatin reorganisation; indeed, the regulation of this histone modification appears molecularly distinct from the initial effects of ERK1/2 dephosphorylation and correlates with later transcriptional onset (Fig 6C–6C” compare Fig 2D–2D”). These distinct steps in regulation of the exemplar gene *PAX6* are summarised in Fig 7.

## Discussion

In this study, we uncover a molecular mechanism by which the neural inducing signal FGF regulates higher-order chromatin organisation to engage the neural differentiation programme. We demonstrate that loss of the activity of FGF-effector kinase ERK1/2 leads to rapid chromatin decompaction at the neural gene *PAX6* in the caudal lateral epiblast of mouse embryos and in analogous hESC-derived NMP-L cells in vitro. Using ATAC-seq, we show that this reflects global remodelling involving increased chromatin accessibility across thousands of neural genes. Focusing on *PAX6* as an exemplar neural progenitor gene, we find that ERK1/2 dephosphorylation results in reduced occupancy by polycomb proteins and that this is not explained by changes in PRC protein levels, although PRC2 protein Jarid2 declines during differentiation. Moreover, ERK1/2 inhibition did not alter ERK2 association with chromatin at this locus. We further show that while *PAX6* is a bivalent gene poised for expression in NMP-L cells, PRC protein loss following ERK1/2 inhibition is distinct from later actions that mediate removal of the gene silencing mark H3K27me3 and transcriptional onset. Importantly, transient ERK1/2 inhibition triggered increased chromatin accessibility and PRC loss, which was not reinstated on resumption of ERK1/2 signalling, and this suggests that a decrease in ERK1/2 activity serves as a gating mechanism that prompts spinal cord differentiation progression. Furthermore, genomic footprinting of sites identified by ATAC-seq together with ChIP-seq for polycomb protein Ring1B revealed that ERK1/2 inhibition increased neural TF occupancy at non-polycomb as well as polycomb associated sites. Together, these findings suggest a model in which FGF/ERK1/2 signalling promotes or maintains PRCs at a subset of neural gene loci while its decline initiates the neural programme by synchronising removal of this and other chromatin remodelling complexes, globally increasing accessibility for neural TFs, which drive differentiation (summarised for exemplar gene *PAX6* in Fig 7).

The ability of ERK1/2 inhibition to elicit chromatin decompaction at the *PAX6* locus in both the mouse embryo and human ESC derived NMP-L cells indicates that the molecular mechanism by which ERK1/2 regulates chromatin accessibility is conserved across species. Establishing *PAX6* as a polycomb target in both contexts further identified this repressive complex as the likely mediator of chromatin compaction. PRC1-generated compaction has been elucidated in mESCs [67–70], and *Pax6* has been identified as a PRC target in this context too [99]. The rapid decompaction of chromatin (within just one hour) following ERK1/2 inhibition in the mouse embryo uncovered here further suggests that ERK1/2 activity may directly maintain PRC occupancy. Exposure of human NMP-L cells to MEKi led to loss of the PRC1/Ring1B and PRC2/Jarid2 by 12 hours at the *PAX6* locus, accompanied by local chromatin decompaction, while in MEKi-treated differentiating NMP-L cells Ring1B was lost, Jarid2 was now almost below detection in all conditions. These findings raise the possibility that PRC1 is the primary target of ERK1/2 activity, while low level Jarid2 at chromatin in differentiating NMP-L cells may reflect global decline in PRC2 proteins during neural differentiation, as documented during mouse ESC differentiation into forebrain neural progenitors [46] and suggested by decreasing levels of Jarid2 detected here by western blot. This is consistent with declining chromatin-associated Jarid2 evident in wild-type conditions, where it is enriched on D3 and D5 and falls below immunoglobulin G (IgG) control levels on D7 and D8 (Figs 2D’ and 3B). Further supporting this, *Jarid2* in mouse embryos is highly transcribed in the CLE and later tailbud and is down-regulated as cells form the neural tube (S1 Fig). However, the finding that in NMP-L cells MEKi exposure does not alter *Jarid2* transcript levels after 12 hours while reducing Jarid2 occupancy across *PAX6* and at *HOXD11* (Fig 6D”) indicates that ERK1/2 activity loss can acutely impact Jarid2 protein availability/association with chromatin. This suggests that decline in ERK1/2 activity may act at multiple levels to attenuate PRC2 at differentiation genes. Indeed, while Jarid2 expression is characteristic of NMPs, it would be interesting to monitor regulation of other PRC2 proteins by this signalling pathway. Moreover, although we found Ring1B protein levels reduced slightly during neural differentiation of NMP-L cells (confirming similar mild down-regulation in mESC differentiation; [46]), ERK1/2 inhibition did not alter Ring1B levels (S3 Fig), indicating that ERK1/2 signalling dynamics do not impact PRC1 occupancy by altering availability of this core protein. Furthermore, we note that while it would be interesting to assess the impact of declining ERK1/2 activity on further PRC1 proteins, we found that MEKi exposure led to global increase in chromatin accessibility at neural genes many of which were Ring1B occupied in NMPs, indicating that even if a Ring1A containing PRC1 complex is present in this context this is not operating to maintain chromatin compaction following loss of Ring1B occupancy. Together, these data using representative PRC 1 and PRC2 proteins suggest that loss of ERK1/2 activity reduces these complexes at differentiation genes by multiple distinct mechanisms; indeed, this may initially impact PRC1, while PRC2 gene/protein levels also decline during differentiation.

A potentially direct mechanism by which ERK1/2 regulates PRC2 has been reported in mESCs, where ERK2 protein associates with PRC2/Jarid2 occupied chromatin [82]. In this study, abrogating ERK1/2 signalling reduced Jarid2 and H3K27me3 at developmental genes, raising the possibility that ERK1/2 protein is directly involved in PRC recruitment and/or maintenance [82]. We found evidence for decline in ERK2 association with chromatin at *PAX6* and *HOXD11* during neural differentiation. However, we also observed ERK2 at the *GAPDH* TSS indicating that in this context, its association with DNA is not PRC target gene specific. Furthermore, inhibition of ERK1/2 activity did not alter ERK2 association with chromatin at these PRC target loci. Our finding of change in PRC occupancy over time may be explained by a decline in ERK1/2 levels during neural differentiation, while experiments involving acute ERK1/2 inhibition suggest that a decrease in ERK1/2 activity and not ERK2 occupancy correlates with reduced PRC in the context of differentiating NMP-L cells. The differences between our findings and those in mESCs [82] might reflect operation of distinct regulatory mechanisms in these very different cellular contexts. Indeed, ERK1/2 inhibition promotes self-renewal in the mouse ES cell state, while loss of FGF and so ERK1/2 promotes differentiation progression of human ESC and NMP-L cells. Moreover, the observation that NMP-L cells exposed to MEKi lose PRC occupancy and decompact the *PAX6* locus, despite resumption of ERK1/2 signalling (in the continued presence of exogenous FGF) indicated that the effects of ERK1/2 inhibition in differentiation conditions are not easily reversed. This contrasts with the findings in mES cells, where rescue of ERK1/2 mutant ESCs with ERK2 restored PRC mark H3K27me3 [82]. Loss of ERK1/2 signalling in NMP-L cells may therefore serve as a gating mechanism that promotes differentiation progression and appears an initial step in the process of neural commitment.

Importantly, in the experiments in which NMP-L cells were exposed to MEKi for 12 hours, chromatin decompaction and loss of both Jarid2 and Ring1B at the *PAX6* locus was not accompanied by change in H3K27me3 levels, and *PAX6* was not transcribed. These findings and those in differentiation conditions at D6 show that the mechanism(s) by which loss of ERK1/2 activity removes PRC are distinct from those that direct subsequent gene transcription. Such distinct regulation of chromatin accessibility is consistent with observations in Ring1B-deficient mES cells, where *HoxB* and *D* loci decompact while their gene promoters remain decorated with H3K27me3 [67]. These findings are also consistent with the retention of H3K27me3 after PRC2 protein binding is lost during mESC neural differentiation [46] and in mouse mutant embryos in which PRC2 genes have been conditionally removed [76,100] Although there are examples of transcription from loci carrying H3K27me3 [40,101], our findings here indicate that H3K27me3 removal is closely temporally associated with the transcriptional derepression of *PAX6* (this mark is not significantly lost until D8) (Figs 2D and 3B). These findings emphasise the value of the temporal resolution afforded by this slow in vitro differentiation assay in which signalling can be manipulated and the molecular mechanisms regulating chromatin accessibility decoupled from those directing transcription initiation.

Using ATAC-seq, we identified genes with increasing accessibility during neural differentiation of NMP-L cells and identified thousands of genes with GO terms associated with neural development. These included *PAX6*, but also other neural progenitor genes (e.g., *SOX1*, *GlI3*, *GlI2*, *RARB*, *NKX6*.*1*, *DBX1*, *NEUROG2*), which commence expression in the mouse and chick embryonic body axis, like *Pax6*, after FGF/ERK1/2 signalling decline [20,22,35]. We compared our “neural sites” (i.e., those that show increased accessibility during differentiation of NMP-L cells (D3) into NP cells (D8)) with the data for the H3K27me3 mark in hESCs available on ENCODE, and this showed only a small overlap (S2 Fig). This suggested that a subset of “neural sites” are PRC targets and that increased accessibility at other sites is regulated either as a downstream consequence of expression of PRC target genes such as *PAX6*, which is known to regulate expression of other neural differentiation TFs, including the master neuronal differentiation gene Neurog2 [102] or by a PRC-independent mechanism. The extent to which these genes are direct PRC targets was then tested by intersection of ATAC-seq-defined D5 and D6 neural sites uniquely induced by MEKi with global sites of Ring1B occupancy in NMP-L cells. An important conclusion from this work is that the deposition of Ring1B alone does not globally define all sites that later change in accessibility upon ERK1/2 signalling decline. Instead, our analysis identified a subset of sites/associated genes that are likely direct PRC targets. This suggests that declining ERK1/2 activity increases chromatin accessibility at neural genes by removing PRC but also affecting other chromatin regulatory machinery. Indeed, genomic footprinting of these MEKi-induced neural sites provided evidence that this event is likely driven by increased SOX family TF engagement at both PRC and non-PRC-associated sites. Moreover, our analysis indicated that SOX family TFs are enriched in neural sites on D5 in MEKi conditions, prior to the time when such treatment leads to precocious *PAX6* transcription on D6, indicating that increased chromatin accessibility and neural TF binding are ERK1/2-regulated steps distinct from the machinery of transcription (Fig 7). A further possibility for the action of declining ERK1/2 activity could be through the regulation of enhancers that in turn activate gene expression and lead to PcG eviction. This further mechanistic possibility could be explored in the future once enhancer sequences and their target promoters have been functionally validated and may reveal not only SOX motifs, but also those for TFs whose activity is directly controlled by ERK1/2, such as ETS factors.

Further ways in which FGF/ERK1/2 signalling decline may direct chromatin reorganisation include alteration of BAF (or SWI/SNF) complex composition, leading to its recruitment and so eviction of PRC proteins from gene loci, as reported in mouse fibroblasts and ESCs [103]. This is an attractive possibility as it might also provide specificity for PRC removal at neural genes, encoded in BAF complex composition [104]. This complex alters as neural progenitor cells become neurons [104,105], while in differentiating NMP-L cells, we would expect loss of ERK1/2 activity to lead to initial formation of the BAF complex characteristic of neural progenitors. In support of such a step, transcription of key BAF complex component the ATP-dependent helicase *SMARCA2* (Brahma homolog) is sharply down-regulated as FGF/ERK1/2 signalling declines in the elongating embryonic body axis [93]. Interesting, this coincides with down-regulation of *Jarid2* and also the histone deacetylase *HDAC1* in the embryo: Moreover, here, *HDAC1* expression depends on FGFR signalling [93]. These observations point to alignment of FGF/ERK1/2 signalling loss and a coordinated change in the expression of genes that contribute to multiple chromatin regulatory complexes as differentiation commences.

The extent to which FGF/ERK1/2 signalling directs progression of differentiation by regulating PRC occupancy in contexts other than caudal epiblast/NMP-L cells requires further investigation. In mouse ESCs maintained in serum and LIF, PRC and H3K27me3 are detected at differentiation gene loci and this constitutes primed pluripotency, characterised by heterogenous cell states in a dynamic flux between pluripotency and differentiation [81,106]. This contrasts with the ground-state or naïve pluripotent cell state achieved by treatment with both ERK1/2 and GSK3beta inhibitors (2i) [29,107], in which differentiation genes lack both PRC and H3K27me3 [81]. Importantly, differentiation of such 2i ES cells involves progression through the primed pluripotent cell state, suggesting that this involves recruitment of PRC and establishment of bivalent loci. Progression towards differentiation involves FGF/ERK1/2 signalling [32,108], but only a transient period is required to initiate this process [31]. This last step is consistent with the differentiation of mEpiSCs and hESCs (for which FGF is a self-renewal factor) when FGF signalling is blocked [34,36]. These observations, together with the requirement for PRC1 and 2 for mESC differentiation [88], indicate a good correlation between exposure to a sufficient period of FGF/ERK1/2 signalling, PRC presence at differentiation genes, and subsequent differentiation from the ES cell state, following ERK1/2 signalling decline. These findings, along with our observation that ERK1/2 activity is required for PRC occupancy at *PAX6* in NMP-L cells and that its inhibition triggers precocious *PAX6* transcription in differentiation conditions, suggest that promotion of PRC at neural genes by ERK1/2 signalling may function as a rite of passage for subsequent differentiation.

In the future, it will be important to investigate whether ERK1/2 signalling dynamics operate to coordinate chromatin accessibility and expression of differentiation genes in other FGF-expressing progenitor cell populations in the developing embryo, including in limb, lung, and tooth buds [109–112], as well as in distinct regions of the nervous system [113]. Intriguingly, differentiation during epidermal homeostasis also requires ERK1/2/MAPK phosphatase DUSP6 in this adult tissue context [114]. This underscores the idea supported here that a transient ERK1/2 signalling dynamic acting on PRC and other chromatin complexes constitutes core and conserved molecular machinery that is a prelude to cellular differentiation.

## Materials and methods

### Embryo dissection and hanging drop culture

All animal husbandry and procedures were approved by the UK Government Home Office and were in accordance with European Community Guidelines (directive 86/609/EEC) under project licence number 6004454. CD1 mouse embryos collected at embryonic stage E8.5 were dissected in warm medium (DMEM-F12 with 10% FBS, Gibco) and one half of the litter exposed to MEK inhibitor (PD184532, MEKi, 3 μM final concentration, Enzo Life Sciences) and the other half exposed to vehicle control DMSO (equal volume, 1:4,000). Each embryo was placed in a drop of medium in the lid of a 35-mm plate, which was flipped over onto its base, resulting in a hanging drop [115]. Embryos were cultured in a humid chamber at 37°C and 5% CO_2_ for 1 hour. For FISH, embryos were fixed immediately after 1 hour in hanging drop culture and processed as detailed below. For ChIP-qPCR, embryos were placed on ice in cold culture medium and the caudal region explants microdissected as shown in Fig 1E, before being collected and fixed with formaldehyde (Sigma-Aldrich, 1%, 10 minutes, RT) and quenched with glycine (0.125 M, 5 minutes, RT) and snap frozen until the ChIP procedure.

### Whole embryo in situ hybridisation

Mouse embryos for whole embryo in situ hybridisation were dissected at required stages (E8.5 to E13.5), fixed overnight in cold paraformaldehyde, and processed using standard protocols. Sequences for the *Jarid2* transcript variants were obtained from the database NCBI (NM_001205043.1, NM_021878.3, and NM_001205044.1), and primers (5′ GAGGAGGAAGAAGACAAA 3′; 5′ CATGGAGAATGGCTTAGC 3′) designed to amplify the region homologous to the sequence used for the *Jarid2* probe in the chicken [93]. The sequence was PCR amplified under standard conditions and annealing temperature of 55°C. The PCR product was purified using the QIAquick Gel Extraction kit (QIAgen, Cat. #28704) following manufacturer’s instructions and cloned into a StrataClone PCR Cloning Vector (pSC-A-amp/kan) (Strata Clone PCR Cloning kit, Agilent Technologies, Cat. #240205–5) according to manufacturer’s instructions. A positive white colony was picked for plasmidial DNA analysis (MaxiPrep—QIAfilter Plasmid Maxi Kit Qiagen, Cat. #12263) and presence of the insert was confirmed by sequencing. The plasmid was then used to generate a probe for *Jarid2* expression analysis in the mouse embryo.

### Cell culture of human ESCs

Work with hESCs was undertaken in approval of the UK Stem Cell Bank steering committee (licence number SCSC14-29). Human ES cells (H9, WiCell) were authenticated by short tandem repeat (STR) DNA profiling. Cell banks were tested for sterility by direct inoculation of conditioned medium into tryptic soya broth and soya bean casein broth, and no contamination was observed; mycoplasma testing was carried out by DAPI staining of fixed cultures and with mycoalert mycoplasma detection kit (Lonza). Cell lines were tested to check for the uniform expression of pluripotency markers (Oct4, Sox2, Nanog, SSEA-3, SSEA-4, TRA-1-60, and TRA-1-81) and absence of differentiation markers (SSEA-1, HNF-3 beta, beta-III-tubulin, and smooth muscle alpha-actinin) by immunofluorescence.

H9 hESCs cells were maintained in TESR medium on Geltrex (Thermo Fisher Scientific)-coated dishes (10 μg cm^−2^) and provided for experiments by the Human Pluripotent Stem Cell Facility of the University of Dundee. For differentiation, cells were plated on Geltrex-coated dishes (20 μg cm^−2^) at a density of 4 × 10^3^ cells cm^−2^ and allowed to attach overnight. To generate NMP-L cells, ES media was removed, and cells were cultured with neurobasal medium (Gibco) supplemented with N2, B27, GlutaMAX (final concentration for each 1×, Gibco), 20 ng/ml bFGF (Peprotech), and 3 μM CHIR99021 (Tocris Bioscience) for 3 days (after [13]). To generate neural progenitor cells (NPs) from these NMP-Ls, bFGF and CHIR99021 were removed from the medium, cells were then cultured from day 2 to day 4 in medium further supplemented with BMP and TGFβ inhibitors (Noggin [50 ng/ml], Peprotech and SB431542 [10 μM], Tocris Bioscience). NMP-L cells were generated on day 3 and this was routinely confirmed by RT-qPCR using primers for BRACHYURY/T and SOX2. NMP-L cells were differentiated into NPs in neurobasal medium supplemented with N2, B27, and GlutaMAX (with day 4 containing BMP and TGFβ inhibitors) for up to day 8, and neural differentiation of posterior NPs was confirmed by RT-qPCR using primers for *PAX6* and *HOXD11*. This protocol is identical to that we describe in [91] to make hNMP-Like cells, but here, differentiation of NMP-L cells into NPs was undertaken without addition of exogenous retinoic acid from day 4 (protocol schematic; Fig 4A). Treatment with MEKi (PD184352 [3 μM], Tocris Bioscience)/DMSO or DMSO alone (equal volume) was administered with the culture medium in a dilution of 1:4,000 for transcript-level analysis and ChIP-qPCR experiments, additional revision experiments reported in S4A–S4F Fig used the MEKi PD032590 at 3 μM or DMSO at 1:4,000, MEKi was introduced in regimes as described in each experiment. In ATAC-seq experiments, PD184352, MEKi /DMSO, or DMSO alone (equal volume) at 1:20,000 was administered with the culture medium.

### Fluorescence in situ hybridisation (FISH)

The mouse *Pax6* fosmid pair (WIBR-1 Mouse Fosmid Library, Whitehead Institute/MIT Center for Genomic Research) and the human *PAX6* BAC clone pair (WIBR2 Human Library; see S4 Table) were prepared using a standard Mini-prep protocol. Using Nick transcription, the fosmids and BAC clones were labelled with Digoxigenin11dUTP and Biotin-16-dUTP. Unincorporated nucleotides were removed with Quick Spin G50 Sephadex columns (Roche) and labelled probes quantified by dot blotting. Embryos were exposed for short term to MEKi or vehicle control DMSO by 1 hour hanging drop culture, then fixed (4% PFA, overnight), washed, and dehydrated through a methanol series before being cleared in xylene and embedded in paraffin for sectioning (7 μm). The FISH protocol on mouse tissue was adapted from [116]. Coverslips with sections containing neural tube or caudal lateral epiblast tissue were heated to 65°C for 30 minutes, then xylene washed (4 × 10 minutes each) and rehydrated through an ethanol series to dH_2_0. The coverslips were microwaved for 20 minutes in 0.1 M citrate buffer (pH 6.0), then left to cool and washed in dH_2_0. For probe hybridisation, 150 ng each of the labelled probes together with 15 μg mouse Cot1 DNA and 5 μg sonicated salmon sperm DNA were denatured and incubated overnight at 37°C. After a series of washes, the probes were detected using FITC-conjugated anti-Digoxigenin antibody (1:20, Roche) amplified with anti-sheep Alexa Fluor 488 (1:100, Molecular Probes) and biotinylated anti-Avidin (1:100) together with Alexa streptavidin 594 (1:500, Molecular Probes); nuclei were counterstained with DAPI, coverslips were mounted with Slowfade Gold (Molecular Probes) and imaged on a Deltavision (Applied Precision).

For FISH on human ESC–derived cells, cells were directly grown and differentiated on glass coverslips coated with Geltrex and cells were untreated, exposed to DMSO or MEKi in DMSO as above. After a short fixation (10 minutes, 4% formaldehyde, RT), the coverslips were washed in 0.05% Triton X100/PBS (3 × 5 minutes, RT). For permeabilisation, the coverslips were incubated in 0.5% Triton X100/PBS for 10 minutes, then transferred to 20% glycerol/PBS for 1 hour at 4°C and then repeatedly dipped in liquid nitrogen until completely frozen, let to thaw, and soaked in 20% glycerol/PBS again (6 repeats). After the last snap freezing, the coverslips were washed in 0.05% Triton X100/PBS (3 × 5 minutes, RT), rinsed and incubated in 0.1 N HCl (10 minutes, RT), and washed in 0.05% Triton x100/PBS (3 × 5 minutes, RT) again. To equilibrate, coverslips were washed in 2xSSC before prehybridisation (50% Formamide in 2xSSC) for 30 minutes at RT. For probe hybridisation, the same probe mix as for the FISH on mouse material was used (except probes specific for the human Pax6 locus). The probes were denatured on a heat block at 75°C for 3 minutes before hybridisation (overnight, 37°C, humid chamber). The coverslips were then washed, and probes were detected using the same antibody reactions as for the FISH on mouse samples; nuclei were counterstained with DAPI, coverslips were mounted with Slowfade Gold and imaged using a widefield Deltavision Microscope. Using OMERO insight regions of interest (ROIs) were selected over at least 3 z-sections. The 3D interprobe distances in ROIs were measured using a custom script called OMERO mtools [117], by segmenting the objects from the background and calculating the distance between the centroids as d in μm. For ease of comparison, the ratio of number of base pairs per nm was calculated using the interprobe distance known in bp and measured in nm.

### Chromatin immunoprecipitation (ChIP)

For each IP and control IP, 25 μL of dynabeads coated with protein A (Invitrogen) were used. Beads were washed 3 times in blocking buffer (0.1% BSA in PBS) and resuspended in blocking buffer containing the antibody for immunoprecipitation or an equal amount of unspecific IgG (for antibody source information, see S5 Table) and incubated for 2 to 3 hours at 4°C. Beads were then washed twice in blocking buffer, resuspended in blocking buffer, and stored at 4°C until immunoprecipitation.

For ChIP on human ES cells, the cells were grown and differentiated in 10 cm culture dishes at 37°C and 5% CO_2_. Cells were fixed with formaldehyde (Sigma-Aldrich, 1%, 10 minutes, RT) and quenched with glycine (0.125 M, 5 minutes, RT). Cells were then washed 3 times with PBS and harvested by scraping into protein low bind Eppendorf tubes and centrifuged (4,000 x *g*, 10 minutes, 4°C). The pellets were snap frozen on dry ice and stored at −80°C. For the chromatin preparation, the cell pellet was resuspended in lysis buffer (50 mM Tris–HCl (pH 8.1); 10 mM EDTA (pH 8); 1% SDS and protease inhibitor), vortexed, and incubated (10 minutes on ice). Chromatin fragments of approximately 500 to 1,000 bp were generated by sonication with a probe sonicator (Vibra-Cell, Sonics 8 cycles, 30 seconds on, 30 seconds off, 30% amplitude). Sonicated lysate was diluted (1:5) in dilution buffer (20 mM Tris–HCl (pH 8.1); 150 mM NaCl; 2 mM EDTA (pH 8) 1% Triton X100 and protease inhibitor) and centrifuged (20 minutes, full speed, 4°C). The supernatant was transferred into a fresh tube. DNA concentration was determined with NanoDrop 2000 Spectrophotometer.

For ChIP of chromatin associated proteins 100 μg of chromatin and ChIP of histone modifications 25 μg of chromatin were diluted to a final volume in 1 mL, added to the already prepared bead-antibody complexes, and incubated (overnight, 4°C, rotating). An input for each ChIP (10% of ChIPed amount) was kept at −20°C. The next day, beads were washed 5 times in wash buffer (100 mM Tris (pH 8.8); 0.5 M LiCl; 1% NP40, 1% NaDoc and protease inhibitor) and then rinsed and washed in 1x TE (10 mM Tris and 1 mM EDTA (pH 8)). To elute, the beads were resuspended in elution buffer (50 mM Tris–HCl (pH 8); 10 mM EDTA (pH 8); 1% SDS) and incubated (15 minutes, 65°C, 1,400 rpm). The eluate was spun down quickly, and the supernatant was transferred into a fresh tube. Formaldehyde crosslinking was reversed by incubation at 65°C and 700 rpm for 6 hours. The input sample was diluted with elution buffer (1:1) and crosslinking reversed. All samples were then diluted with TE (1:1), and RNA and proteins were digested (RNAse, Sigma-Aldrich, 30 minutes, 37°C and proteinase K, Sigma-Aldrich, 90 minutes 45°C). DNA was recovered by Phenol/Chloroform extraction (Phenol:Chloroform:Isoamylalcohol, 25:24:1 (pH 8), Sigma-Aldrich) and washed with Chloroform:Isoamylalcohol (24:1, Amresco) and then precipitated in 96% ice cold ethanol, with 3 M SodiumAcetate and linear polyacrylamide (5 mg/mL, Ambion) overnight at 80°C. Precipitated DNA was washed with 70% ice cold ethanol, and pellet was air dried before resuspension in nuclease free water.

A similar protocol was used to ChIP material from caudal mouse explants. Thirty fixed and snap frozen caudal explants were pooled and resuspended in lysis buffer, vortexed, and dissociated by pipetting before incubating on ice for 10 minutes and sonication. The lysate was diluted in dilution buffer, centrifuged (20 minutes, maximum speed, 4°C), and the supernatant was transferred into a new tube (and the DNA concentration determined by NanoDrop 2000 Spectrophotometer 0.25 μg of chromatin were used to immunoprecipitate with 3 μg of the rabbit anti H3K27me3 antibody (Millipore) previously bound to dynabeads overnight at 4°C). After that, the same procedure of washing, eluting, uncrosslinking, and precipitation of DNA was used. For quantification of specifically immunoprecipitated DNA, quantitative real-time PCR was performed (PerfeCTa SYBR Green SuperMix for iQ, Quanta Biosciences or Brilliant III Ultra-Fast QPCR Master Mix, Agilent Technologies). Each sample was analysed in 3 biological replicates, and each biological replicate was analysed in technical triplicates using primer pairs in S6 Table.

### Transcription-level analysis by reverse transcription quantitative PCR (RT-qPCR)

For RNA extraction, human ES cells were grown and differentiated in 24-well plates. Cells were washed with PBS before being collected in 350 μL of lysis buffer from the Qiagen RNeasy Mini Kit, and RNA was prepared according to manufacturer’s protocol. An on-column DNA digest with RQ1 RNase-Free DNase (Promega) was performed (RT, 15 minutes). cDNA was generated from 500 μg purified RNA with the ImProm-II Reverse Transcription System (Promega) primed by random primers. The Aria Mx real-time PCR system with the Brilliant III Ultra-Fast QPCR Master Mix (Agilent Technologies) was used to quantify the transcript levels. Each sample was analysed in biological triplicate, and each biological replicate was run in technical triplicates (for primers, see S7 Table). The relative transcription level of a gene was normalised to that of *GAPDH* using the Pfaffl method [118], and *GAPDH* levels were checked by normalisation to *HPRT1*.

### Western blotting

A minimum of 4 stage E8.5 mouse embryos or human ES cells grown in a 6-well plate were lysed in lysis buffer (LB Nuc+ all), kept on ice for 30 minutes while vortexing every 5 minutes, and centrifuged (16,000 x *g*,4°C, 20 minutes). The supernatant was transferred into a new tube and protein concentration determined by Bradford assay. Approximately 50 μg of protein were separated on a 4% to 12% precast Gel (Invitrogen) in 1x MES buffer (Invitrogen) next to a size marker before blotting on onto a PVDF membrane in transfer buffer (25 mM Tris, 190 mM glycine, 20% methanol). To minimise variances, every sample was loaded at least twice on the same gel and blotted onto a membrane at the same time. After blotting the membrane halved and one half was processed with a pan antibody and an antibody for the loading control α-Tubulin and the other half with a phosphorylation specific antibody and the loading control antibody. Membranes were first blocked in 3% BSA in TBST for at least 2 hours at RT, before incubation with primary antibody (S8 Table) overnight. After washing in TBST (3 × 10 minutes, RT), the membrane was incubated with secondary antibodies (see S8 Table; 90 minutes, RT). Following TBST washes, signals were visualised by scanning on LICOR Odyssey system. Band intensity measurements were performed for quantification. The loading controls were used to normalise for minor differences in loading before the phosphorylated ERK1/2 or PKB levels were compared to the pan ERK1/2 and PKB levels of the same samples. Ring1B and Jarid2 western blotting was performed as described above except HRP-conjugated anti-species secondary antibodies were used and proteins were detected using enhanced chemiluminescence (ECL, GE Healthcare) and exposure to X-ray film. Anti-beta-actin and anti-GAPDH antibodies were used as loading controls. For antibody source information, see S8 Table and S1 Raw Images for western blot images.

### ATAC-seq

#### Experimental procedure

ATAC-seq was performed on NMP-L cells, D5 and D6 cells in untreated, DMSO-treated, or MEKi-treated condition, and NP (D8) cells with the following modifications to the method described by Buenrostro and colleagues [94,119]. Cells were treated with TrypLE Select (Thermo Fisher) to obtain a single-cell suspension. Cells were then resuspended and counted in ice cold PBS and 50,000 cells per sample were used for the ATAC protocol (biological replicates were collected from independent experiments). Cells were pelleted and resuspended in lysis buffer (10 mM Tris–HCl (pH 7.4), 10 mM NaCl, 3 mM MgCl_2_, 0.1% NP-40) and centrifuged (10 minutes, 4°C). Nuclei extracts were resuspended in transposition buffer for 4 hours at 37°C. The reaction was purified using the Qiagen MinElute PCR Purification kit according to manufacturer’s instructions. To generate single-indexed libraries, the transposed DNA was PCR amplified with Nextera primers [94]. Library quality was assessed using Agilent TapeStation D5000 High Sensitivity Screen Tapes or a High Sensitivity DNA analysis kit for the Bioanalyzer.

#### ChIP-seq library preparation

Samples were processed as previously described in [120]. Briefly, hES cells were seeded onto 150 mm diameter dishes and differentiated to NP-like cells as described above. The cells were then washed three times with room temperature Dulbecco’s PBS containing Ca^2+^ and Mg^2+^ (Gibco) and proteins cross-linked with 2 mM disuccinimidyl glutarate (DSG, Pierce) in Dulbecco’s PBS with Ca^2+^ and Mg^2+^ for 45 minutes at room temperature with gentle agitation. Cells were then washed three times with Dulbecco’s PBS (Gibco) and DNA cross-linked with 1% methanol-free formaldehyde (Sigma Aldrich) in Dulbecco’s PBS for 10 minutes at room temperature with gentle agitation. The formaldehyde was then quenched by adding glycine to a final concentration of 0.25 mM and incubating for 5 minutes at room temperature. The cells were then washed twice in Dulbecco’s PBS and harvested by scraping into Dulbecco’s PBS containing 0.02% (w/v) Triton X-100 on ice and collected by centrifugation at 500 x *g* for 5 minutes at 4°C. The supernatant was then removed using a pipette and the cell pellet frozen in liquid N_2_ and stored at −80°C.

Frozen pellets were thawed on ice, resuspended with a p200 in ice-cold shearing buffer (1% Triton X-100; 0.15 M NaCl; 1 mm EDTA; 0.5 mM EGTA; 20 mM HEPES (pH 7.6)) with 0.3% SDS and protease inhibitors (Sigma) and transferred to a diagenode tube. Chromatin was sheered using the Diagenode Bioruptor Plus (25 cycles with 30 seconds on/ 30 seconds off; high setting). Lysates were diluted to 0.15% SDS and cleared by centrifugation at 14,000 rpm for 10 minutes at 4 degrees. Around 1/20th of the lysate was snap frozen in liquid nitrogen and set aside for use as the input. The remaining lysate was incubated on a rotating wheel overnight at 4 degrees with 100 μl of protein G dynabeads (Invitrogen) that had been preloaded for 4 hours at room temperature with 2 μg ChIP-grade Ring1B antibody (MBL D139-3) diluted in sheering buffer with 0.15% SDS. Beads were immobilised using a magnet, and unbound supernatant discarded. Beads were sequentially washed under rotation twice with wash buffer 1 (0.1% SDS; 0.1% deoxycholate; 1% Triton X-100; 0.15 M NaCl; 1 mM EDTA; 0.5 mM EGTA; 20 mM HEPES (pH 7.6)), once with wash buffer 2 (0.1% SDS; 0.1% sodium deoxycholate; 1% Triton X-100; 0.5 M NaCl; 1 mM EDTA; 0.5 mM EGTA; 20 mM HEPES (pH 7.6)), once with wash buffer 3 (0.25 M LiCl; 0.5% sodium deoxycholate; 0.5% NP-40; 1 mM EDTA; 0.5 mM EGTA; 20 mM HEPES (pH 7.6)) and twice with wash buffer 4 (1 mM EDTA; 0.5 mM EGTA; 20 mM HEPES (pH 7.6)) for 5 minutes each. Beads were captured using the magnet between each subsequent wash. Chromatin was eluted from the beads by incubating twice at 65 degrees for 10 minutes in 100 μl elution buffer (1% SDS; 0.1 M NaHCO3) on a shaking heating block. Both eluted fractions were pooled together. Input samples were topped up to 200 μl with elution buffer. Both input and ChIP samples were decrosslinked overnight at 65 degrees with 6.4 μl of 5 M NaCl added to each sample. Samples were incubated for 2 hours at 37 degrees with 0.2 mg/ml PureLink RNAse A (Invitrogen), then 5 mM EDTA and incubated for a further 2 hours at 45 degrees with 0.2 μg/ml proteinase K (Thermo Scientific) before purifying the template DNA for library prep using the Qiagen PCR kit following manufacturer’s instructions. Samples were assessed on the Agilent TapeStation prior to library construction using the NEB Ultra II DNA. Biological triplicates were obtained for all samples from separate experiments. Libraries were generated using the Kapa HyperPrep Kit and sequenced as single-end 76 bp reads on the Illumina HiSeq 4000 (The Francis Crick Institute).

### ATAC-seq data analysis

#### Library-level analysis

Sequencing was performed by multiplexing 4 samples per lane on the Illumina HiSeq 2500 platform (The Francis Crick Institute). This typically generated approximately 86 million 51 bp or 101 bp paired-end reads per library. Raw reads from each sample were adapter-trimmed using cutadapt (version 1.9.1) [121] with parameters “-a CTGTCTCTTATA -A CTGTCTCTTATA—minimum-length = 25 –quality-cutoff = 20”. If required, the additional parameters “-u <trim_length>” and “-U <trim_length>” were provided to cutadapt in order to trim all reads to 51 bp. BWA (version 0.6.2) [122,123] with default parameters was used to perform genome-wide mapping of the adapter-trimmed reads to the human hg19 genome assembly downloaded from the UCSC [124]. Read group addition, duplicate marking, and insert size assessment were performed using the picard tools AddOrReplaceReadGroups, MarkDuplicates, and CollectMultipleMetrics, respectively (version 2.1.1) (http://broadinstitute.github.io/picard). Reads mapped to mitochondrial DNA were removed using the pairToBed command from BEDTools (version 2.26.0-foss-2016b) [125]. Additional filtering was performed to only include uniquely mapped, properly-paired reads with insert size < = 2 kb and mismatches < = 1 in both reads. Samtools (version 1.3.1) [122] was used for bam file sorting and indexing. The filtered alignments from each library were merged at both the replicate and sample level using the picard MergeSamFiles command. Duplicate marking and removal were reperformed on the merged alignments. BedGraph coverage tracks representing the accessibility signal per million mapped reads were generated using BEDTools genomeCoverageBed with the parameters “-bg -pc -scale <SCALE_FACTOR>”. BedGraph files were converted to bigWig using the wigToBigWig binary available from the UCSC with the "-clip" parameter [126].

#### Sample-level analysis

To define peaks, regions of chromatin accessibility were identified genome-wide using MACS2 callpeak (version 2.1.1.20160309) [127] with the parameters “—gsize = hs—keep-dup all–f BAMPE—nomodel—broad”. A consensus set of intervals were obtained by merging the regions identified across all samples.

#### Replicate-level analysis

Fragment-level BED files were derived from those created using the BEDTools bamToBed command with the option “-bedpe”. Differential chromatin accessibility sites between conditions were obtained using diffReps (version 1.55.4) [128] with the parameter “—frag 0”. The annotatePeaks.pl program from HOMER (version 4.8) [129] was used to annotate the differential sites relative to the nearest gene promoter with respect to hg19 RefSeq features downloaded from the UCSC on 7 June 2017. Differential sites for each comparison were defined as those that intersected with the consensus set of accessibility sites, had an FDR < = 0.01 and fold-change > = 2, and an annotated gene symbol regardless of distance to TSS. Differential sites that were not allocated a gene symbol were not carried forward for further analysis. Genes associated with differential chromatin accessible regions as identified with ATAC-seq were used as input for GO analysis using https://go.princeton.edu/cgi-bin/GOTermFinder. ATAC-seq metaprofiles were generated using deepTools (version 2.5.3) [130] with the "computeMatrix scale-regions" and "plotHeatmap" commands, respectively. The genome annotation was generated by taking the minimum and maximum genomic coordinates among all the isoforms for a given gene. Raw ATAC-seq data and normalised replicate-level bigWig files have been deposited in the NCBI Gene Expression Omnibus (GEO) under accession code GSE121126) (access token provided to the journal editor).

#### TOBIAS analysis of ATAC-seq data

Genomic footprinting was performed using merged replicate BAM files as input data into the TOBIAS footprinting tool (version 0.12.10; [85]). Analyses were run using the briscoe-nf-tobias pipeline (version v1.1; https://github.com/luslab/briscoe-nf-tobias) written in the Nextflow domain-specific language (version 21.04.0; [131]).

#### ChIP-seq data analysis

The nf-core/chipseq pipeline (version 1.2.1; [132]) (https://doi.org/10.5281/zenodo.3966161), written in the Nextflow domain-specific language (version 19.10.0; [131]) was used to perform the primary analysis of the samples in conjunction with Singularity (version 2.6.0; [133]). The command used was "nextflow run nf-core/chipseq—input design.csv—genome hg19—gtf refseq_genes.gtf—single_end—min_reps_consensus 2 -profile crick -r 1.2.1". All data were processed relative to the human UCSC hg19 genome [124] downloaded from AWS iGenomes (https://github.com/ewels/AWS-iGenomes). Gene annotation files in GTF format were originally downloaded from UCSC on 7 June 2017. For a condensed overview of the pipeline, the bioinformatics tools used in each step, and an extensive list of citations, please see the pipeline homepage. Differential ChIP-seq peaks (FDR 0.01) detected between day 0 and day 3 from three independent biological replicates using [134] were subject to GO analysis using GREAT v4.0.4 [135]. Peak intersections between the ChIP-seq and ATAC-seq data were performed with the BEDTools “intersectBed” command (version 2.26.0-foss-2016b) [125] with the “-u” parameter set and subsequently reannotated with the annotatePeaks.pl program from HOMER (version 4.8) [129] relative to hg19 RefSeq features downloaded from the UCSC on 7 June 2017.

## Supporting information

S1 Fig Jarid2 transcripts detected by mRNA in situ hybridisation in mouse embryos. (**A**) Whole E8.5 embryo, the CLE is where NMPs are located, (**A’**) TS. Jarid2 expression continues (**B**) at E9.5, including in the NMP and for a time in NPs generated by this cell population, shown TS (**B’**, **B”**). This pattern of expression persists (**C**) at E10.5, shown in TS in (**C’**), and is then lost as the tailbud is depleted and axis elongation declines from (**D**) E11.5 and (**E**) 12.5, and ceases at (**F**) E13.5. Scale bars = 100 μm in A, 200 μm in B, C, D-F. CLE, caudal lateral epiblast; NMP, neuromesodermal progenitor; NP, neural progenitor; TS, transverse section.
(PDF)

S2 Fig Neural sites identified by ATAC-seq overlap with known active enhancer sites in human embryonic neural tissue and in vitro generated neural progenitors. Comparison of peaks called from publicly available ChIP-seq data sets for H3K9me3, H3K27me3, H3K4me1, and H3K27ac from the ENCODE regulatory element database [95] with neural sites from Fig 3 show high proportion of overlap for active enhancer marks H3K4me1 and H3K27ac in neural embryonic tissue (brain and spinal cord) and in vitro generated neural progenitors. Data sets from embryonic lung thymus and kidney tissues were used as controls and showed smaller proportions of overlap. Furthermore, H3K9me3 and H3K27me3 were used as control for repressive chromatin modification; H3K9me3 especially is known to be enriched in heterochromatin [136] and shows little to no overlap with the neural sites (for numerical data, see S4 Data).
(PDF)

S3 Fig ChIP-PCR for Jarid2 on Day 6 and western blot for Jarid2 during differentiation. (**A**) ChIP-PCR for Jarid2 for untreated/WT, DMSO, and MEKi-exposed cells during differentiation from NMP-L (D3) to D6 (*n* = 3 independent experiments). These experiments provided low % input and just enriched for WT above IgG control, *t* test shows no significant difference between WT and DMSO or DMSO and MEKi conditions, although WT and MEKi are significantly different, error bars = SEM, * = *p* ≤ 0.05 (S6 Data). These data may reflect the low Jarid2 protein associated with chromatin on D6. (**B**) To assess levels of Jarid2 protein as hESCs differentiate into NMP-L cells and then NPs, we collected protein lysates and ran western blots using antibodies against Jarid2 and GAPDH. This is a sample blot showing one biological replicate and its 3 technical replicates. Bradford assays were used to determine protein concentration and 50 μg protein loaded, note both Jarid2 and GAPDH appear to reduce levels as hESCs differentiate. hESC, human ESC; IgG, immunoglobulin G; MEKi, MEK inhibitor; NMP-L, NMP-like; WT, wild type.
(PDF)

S4 Fig Analysis of global Ring1B levels and ERK2 occupancy during neural differentiation and following exposure to MEKi. To assess levels of Ring1B protein as hESCs differentiate into NMP-L cells and then NPs, we collected protein lysates from 3 independent differentiations and ran western blots, each with three technical replicates using antibodies against Ring1B and beta-Actin. Levels of Ring1B were normalised to b-Actin. (**A**) One biological replicate and its 3 technical replicates (blots for all 3 biological replicates for Ring1B and b-Actin are provided as metadata), and (**B**) quantification of these data. Data analysed using the Student *t* test, error bars ± SD, each dot represents a single data point, *p* = *p < 0.05, ***p < 0.001; NMP-L cells were differentiated towards neural progenitors in control (DMSO only) or MEKi (PD032590, 3 μM)/DMSO conditions for 3 days (assessed on D6) in 3 independent differentiations, replicates 1,2,3,; (**C**, **D**) exposure to MEKi lead to increased expression of PAX6 in all three replicates detected by RT-qPCR (*p* = 0.0024); western blot of 2 technical replicates from each of 3 biological replicates (**E**, **E’**) confirm dephosphorylation of ERK1/2 with antibodies to p-ERK1/2 and compared with total ERK1/2 levels and show (**F**, **F’**) Ring1B and GAPDH protein levels were unaffected by loss of ERK signalling; (**G**) ChIP-qPCR detecting ERK2 occupancy at PAX6 and control loci during differentiation (NMP-L (D3), D5, and D6: black, dark grey, and light grey, respectively) (*n* = 3 independent experiments, error bars = SEM, * = *p* ≤ 0.05, *t* test, comparison between D3 and D6 is significant); (**H** and **H’**) ChIP-qPCR investigating ERK2 occupancy at the PAX6 locus on D5 and D6 of the differentiation protocol comparing untreated, DMSO, and MEKi-exposed samples (*n* = 3 independent experiment, error bars = SEM, no significant differences between treatments, *t* test, MEKi not enriched over IgG on D5 and D6). All underlying numerical data in this figure can be found in S7 Data. ChIP-qPCR, chromatin immunoprecipitation quantitative PCR; hESC, human ESC; MEKi, MEK inhibitor; NMP-L, NMP-like; NP, neural progenitor; RT-qPCR, reverse transcription quantitative PCR; WT, wild type.
(PDF)

S5 Fig Brief ERK1/2 dephosphorylation in NMP-L cells does not alter PRC occupancy, H3K27me3 levels, or transcription. (**A**) Differentiation protocol used to generate NMP-L (D3) cells and treatment regime with vehicle control DMSO or MEKi for 3 hours; (**B**-**B’**) representative western blot of cell lysates probed with antibodies against total (panERK1/2) and dual-phosphorylated-ERK1/2 (dpERK1/2) and LiCOR quantification data (*n* = 3 independent experiments, error bar = SEM, * *p* = < 0.05); (**C**-**E**) ChIP-qPCRs investigating Jarid2 and Ring1B occupancy and H3K27me3 levels at PAX6 and control regions in NMP-L (D3) cells treated with MEKi or DMSO for 3 hours (*n* = 3 individual experiments, bar = average, no significant differences between samples, *t* test), note low Jarid2 input, not enriched over IgG; (**F**-**F”**) transcription levels of PAX6, HOXD11, and JARID2 assessed by RT-qPCR in undifferentiated cell (hESCs), untreated, vehicle control (DMSO)-treated, or MEKi-treated NMP-L (D3) cells (*n* = 3 individual experiments, no significant differences between samples, *t* test). All underlying numerical data in this figure can be found in S9 Data. ChIP-qPCR, chromatin immunoprecipitation quantitative PCR; hESC, human ESC; IgG, immunoglobulin G; MEKi, MEK inhibitor; NMP-L, NMP-like; PRC, polycomb repressive complex; RT-qPCR, reverse transcription quantitative PCR.
(PDF)

S6 Fig NMP-L cells cultured for 12 hours in MEKi no longer exhibit reduced ERK phosphorylation but have increased PKB phosphorylation levels. (**A** and **B**) Western blot analysis of NMP-L (D3) protein extracts using phospho-specific antibodies against ERK1/2 and PKB alongside pan antibodies. (**C** and **D**) Quantification of band intensity shows no reduction in ERK phosphorylation but increased levels of PKB phosphorylation (*n* = 3 independent experiments error bars = SEM, * = *p* ≤ 0.05 or *t* test showed no significant difference). Underlying numerical data for western blots in this figure can be found in S11 Data. MEKi, MEK inhibitor; NMP-L, NMP-like.
(PDF)

S1 Table “Neural sites,” representing a set of genomic intervals and associated genes opening during in vitro neural differentiation identified by ATAC-seq. During in vitro neural differentiation from NMP-L (D3) to NP (D8) 7,877 regions open up associated with 4,001 genes. Regions of increased accessibility are defined by chromosomal position and their associated gene names have been identified by proximity. In the text, we refer to these as “neural sites.”
(XLSX)

S2 Table List of genes associated with open chromatin regions at different time points during in vitro neural differentiation. During in vitro neural differentiation, ATAC-seq reveals that an increasing number of neural genes appear that are associated with open chromatin (as defined by proximity). By day 5 of differentiation, regions associated with 1,143 genes display increased accessibility by day 6 this increases to regions associated with 2,137 genes are open and by final day 8 regions associated with 4,001 genes are now more accessible.
(XLSX)

S3 Table List of genes associated with open chromatin regions comparing control and MEK inhibition conditions on day 5 and day 6 of the in vitro neural differentiation. (XLSX)

S4 Table Codes, coordinates, size, and interprobe distances of mouse fosmids and human BAC clones. (XLSX)

S5 Table Antibodies and amounts used for chromatin immunoprecipitation. (XLSX)

S6 Table Sequences of primers used to analyse chromatin immunoprecipitated DNA. (XLSX)

S7 Table Sequences of primers used for quantification of transcripts by RT-qPCR. (XLSX)

S8 Table Primary and secondary antibodies used for western blotting. (XLSX)

S1 Data Numerical data for: Fig 1C Western data; Fig 1D” FISH interprobe distance data; Fig 1E’ ChIP data. (XLSX)

S2 Data Numerical data for: Fig 2B–2B”’ RT-qPCR data; Fig 2C” FISH interprobe distance data; Fig 2D–2D” ChIP data. (XLSX)

S3 Data Numerical data for: Fig 3B–3B” ChIP data; Fig 3C and 3C’ metaprofile data; Fig 3E GO term data. (XLSX)

S4 Data Numerical data for: S2 Fig Neural sites data and ENCODE data sets. (XLSX)

S5 Data Numerical data for: Fig 4C RT-qPCR data; Fig 4D–4D’ ChIP data; Fig 4F and 4F’ GO term data. (XLSX)

S6 Data Numerical data for: S3 Fig ChIP data. (XLSX)

S7 Data Numerical data for: S4B Fig Western data; S4C and S4D Fig RT-qPCR data; S4G and S4H and S4H’ Fig ChIP data. (XLSX)

S8 Data Numerical data for: Fig 5A and 5B footprint data; Fig 5C, 5D, and 5D’ GO term data. (XLSX)

S9 Data Numerical data for: S5B’ Fig Western data; S5C–S5E Fig ChIP data; S5F-S5F” Fig RT-qPCR data. (XLSX)

S10 Data Numerical data for: Fig 6E’ FISH interprobe distance data. (XLSX)

S11 Data Numerical data for: S6C and S6D Fig Western data. (XLSX)

S1 Raw Images Images of complete blots for all Western data. (PDF)

## Abbreviations

ATAC-Seq: assay for transposaseaccessible chromatin sequencing
ChIP: chromatin immunoprecipitation
CLE: caudal lateral epiblast
DSG: disuccinimidyl glutarate
ECL: enhanced chemiluminescence
ES: embryonic stem; FGF, fibroblast growth factor
FISH: fluorescent in situ hybridisation
GEO: Gene Expression Omnibus
GO: gene ontology
IgG: immunoglobulin G
LB: lysis buffer
MEKi: MEK inhibitor
mEpiSC: mouse epiblast stem cell
NMP: neuromesodermal progenitor
NP: neural progenitor
PcG: Polycomb Group
PRC: polycomb repressive complex
ROI: region of interest
STR: short tandem repeat
TF: transcription factor
TSS: transcription start site
