# Supplementary material for: ERK1/2 signalling dynamics promote neural differentiation by regulating chromatin accessibility and the polycomb repressive complex
Source: PLoS Biol. 2022 Dec 1;20(12):e3000221. doi: 10.1371/journal.pbio.3000221 (PMC9746999; doi:10.1371/journal.pbio.3000221)
Supplement: S1 Fig — (A) Whole E8.5 embryo, the CLE is where NMPs are located, (A’) TS. Jarid2 expression continues (B) at E9.5, including in the NMP and for a time in NPs generated by this cell population, shown TS (B’, B”). This pattern of expression persists (C) at E10.5, shown in TS in (C’), and is then lost as the tailbud is depleted and axis elongation declines from (D) E11.5 and (E) 12.5, and ceases at (F) E13.5. Scale bars = 100 μm in A, 200 μm in B, C, D-F. CLE, caudal lateral epiblast; NMP, neuromesodermal progenitor; NP, neural progenitor; TS, transverse section. (PDF) [file pbio.3000221.s001.pdf]

Supplementary Figures Semprich et al

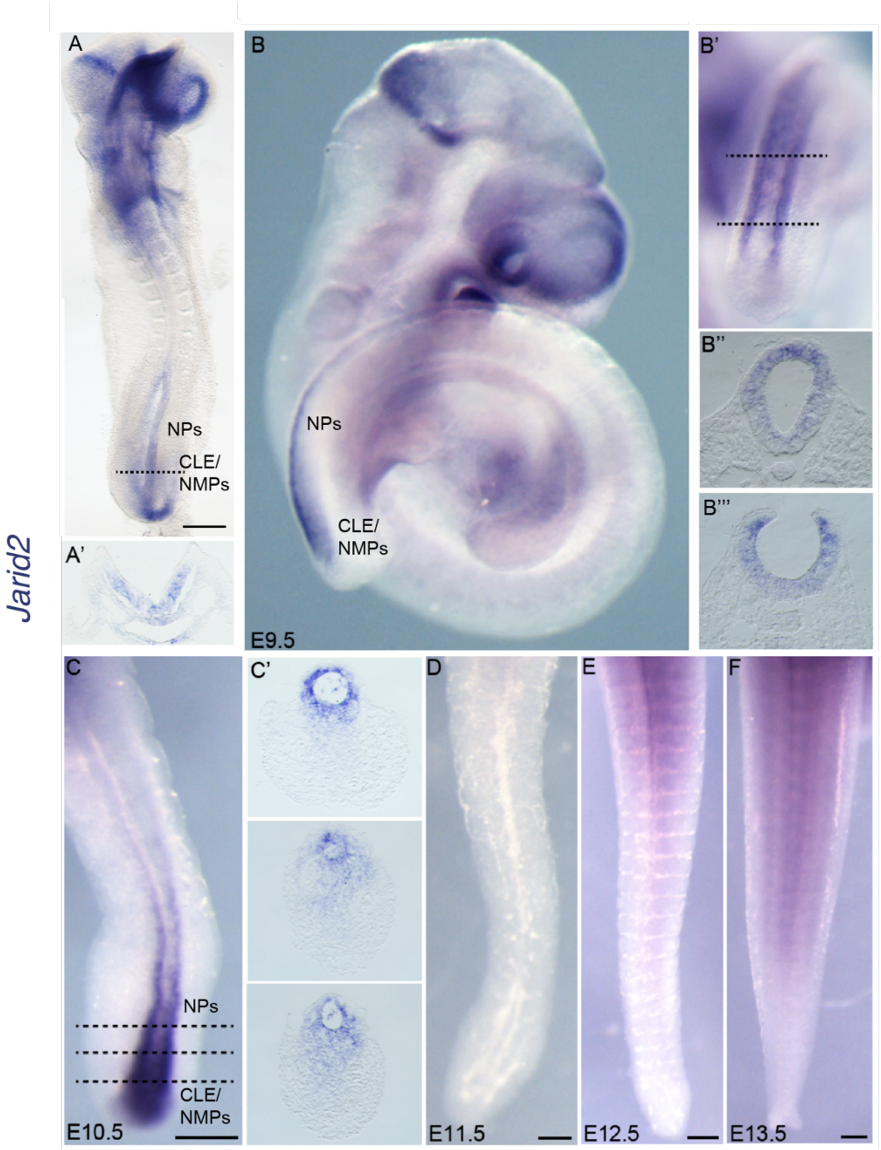

S1_Fig *Jarid2* transcripts detected by mRNA in situ hybridisation in mouse embryos

(A) whole E8.5 embryo, the caudal lateral epiblast (CLE) is where neuromesodermal progenitors (NMPs) are located, A' transverse section (TS). *Jarid2* expression continues (B) at E9.5, including in the NMP and for a time in NPs generated by this cell population, shown TS (B', B''). This pattern of expression persists (C) at E10.5, shown in TS in (C'); and is then lost as the tailbud is depleted and axis elongation declines from (D) E11.5 and (E) 12.5, and ceases at (F) E13.5. Scale bars = 100 µm in A, 200 µm in B, C, D-F.
