# Supplementary material for: ERK1/2 signalling dynamics promote neural differentiation by regulating chromatin accessibility and the polycomb repressive complex
Source: PLoS Biol. 2022 Dec 1;20(12):e3000221. doi: 10.1371/journal.pbio.3000221 (PMC9746999; doi:10.1371/journal.pbio.3000221)
Supplement: S2 Fig — Comparison of peaks called from publicly available ChIP-seq data sets for H3K9me3, H3K27me3, H3K4me1, and H3K27ac from the ENCODE regulatory element database [95] with neural sites from Fig 3 show high proportion of overlap for active enhancer marks H3K4me1 and H3K27ac in neural embryonic tissue (brain and spinal cord) and in vitro generated neural progenitors. Data sets from embryonic lung thymus and kidney tissues were used as controls and showed smaller proportions of overlap. Furthermore, H3K9me3 and H3K27me3 were used as control for repressive chromatin modification; H3K9me3 especially is known to be enriched in heterochromatin [136] and shows little to no overlap with the neural sites (for numerical data, see S4 Data). (PDF) [file pbio.3000221.s002.pdf]

Supplementary Figures Semprich et al

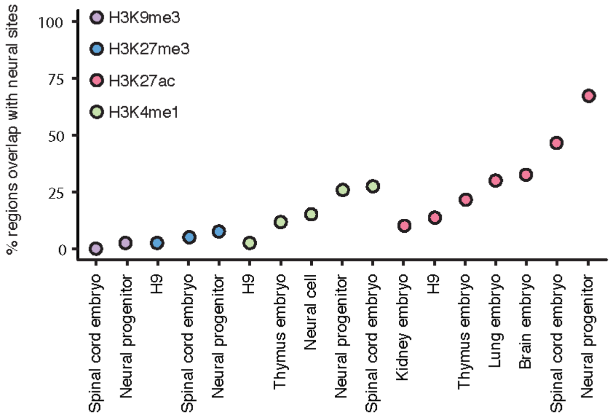

S2_Fig Neural sites identified by ATAC-seq overlap with known active enhancer sites in human embryonic neural tissue and *in vitro* generated neural progenitors

Comparison of peaks called from publicly available ChIPseq data sets for H3K9me3, H3K27me3, H3K4me1 and H3K27ac from the ENCODE regulatory element database [1] with neural sites from Figure 3 show high proportion of overlap for active enhancer marks H3K4me1 and H3K27ac in neural embryonic tissue (brain and spinal cord) and *in vitro* generated neural progenitors. Data sets from embryonic lung thymus and kidney tissues were used as controls and showed smaller proportions of overlap. Furthermore, H3K9me3 and H3K27me3 were used as control for repressive chromatin modification, H3K9me3 especially is known to be enriched in heterochromatin [2] and shows little to no overlap with the neural sites.

1. Sloan CA, Chan ET, Davidson JM, Malladi VS, Strattan JS, Hitz BC, et al. ENCODE data at the ENCODE portal. Nucleic Acids Res. 2016;44(D1):D726-32. Epub 2015/11/04. doi: 10.1093/nar/gkv1160. PubMed PMID: 26527727; PubMed Central PMCID: PMCPMC4702836.

2. Becker JS, Nicetto D, Zaret KS. H3K9me3-Dependent Heterochromatin: Barrier to Cell Fate Changes. Trends in genetics : TIG. 2016;32(1):29-41. doi: 10.1016/j.tig.2015.11.001. PubMed PMID: 26675384; PubMed Central PMCID: PMC4698194.
