# Supplementary material for: ERK1/2 signalling dynamics promote neural differentiation by regulating chromatin accessibility and the polycomb repressive complex
Source: PLoS Biol. 2022 Dec 1;20(12):e3000221. doi: 10.1371/journal.pbio.3000221 (PMC9746999; doi:10.1371/journal.pbio.3000221)
Supplement: S3 Fig — (A) ChIP-PCR for Jarid2 for untreated/WT, DMSO, and MEKi-exposed cells during differentiation from NMP-L (D3) to D6 (n = 3 independent experiments). These experiments provided low % input and just enriched for WT above IgG control, t test shows no significant difference between WT and DMSO or DMSO and MEKi conditions, although WT and MEKi are significantly different, error bars = SEM, * = p ≤ 0.05 (S6 Data). These data may reflect the low Jarid2 protein associated with chromatin on D6. (B) To assess levels of Jarid2 protein as hESCs differentiate into NMP-L cells and then NPs, we collected protein lysates and ran western blots using antibodies against Jarid2 and GAPDH. This is a sample blot showing one biological replicate and its 3 technical replicates. Bradford assays were used to determine protein concentration and 50 μg protein loaded, note both Jarid2 and GAPDH appear to reduce levels as hESCs differentiate. hESC, human ESC; IgG, immunoglobulin G; MEKi, MEK inhibitor; NMP-L, NMP-like; WT, wild type. (PDF) [file pbio.3000221.s003.pdf]

Supplementary Figures Semprich et al

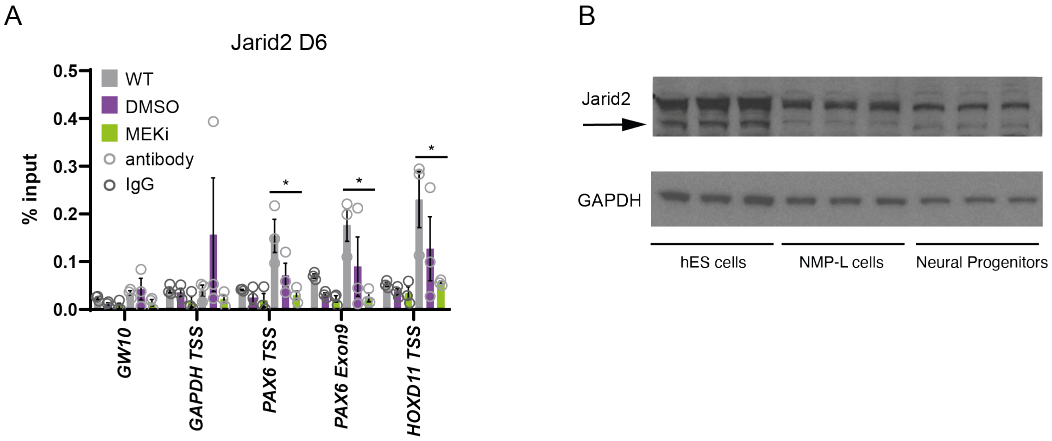

Figure S3 ChIP-PCR for Jarid2 on Day 6 and Western blot for Jarid2 during differentiation

(A) ChIP-PCR for Jarid2 for untreated/WT, DMSO and MEKi exposed cells during differentiation from NMP-L (D3) to D6 (n = 3 independent experiments). These experiments provided low % input and just enriched for WT above IgG control, t-test shows no significant difference between WT and DMSO or DMSO and MEKi conditions, although WT and MEKi are significantly different, error bars = SEM, * = p ≤ 0.05 (S6_data). These data may reflect the low Jarid2 protein associated with chromatin on D6. (B) To assess levels of Jarid2 protein as hESCs differentiate into NMP-L cells and then NPs we collected protein lysates and ran Western blots using antibodies against Jarid2 and GAPDH. This is a sample blot showing one biological replicate and its 3 technical replicates. Bradford assays were used to determine protein concentration and 50 µg protein loaded, note both Jarid2 and GAPDH appear to reduce levels as hESCs differentiate.
