# Supplementary material for: ERK1/2 signalling dynamics promote neural differentiation by regulating chromatin accessibility and the polycomb repressive complex
Source: PLoS Biol. 2022 Dec 1;20(12):e3000221. doi: 10.1371/journal.pbio.3000221 (PMC9746999; doi:10.1371/journal.pbio.3000221)
Supplement: S4 Fig — To assess levels of Ring1B protein as hESCs differentiate into NMP-L cells and then NPs, we collected protein lysates from 3 independent differentiations and ran western blots, each with three technical replicates using antibodies against Ring1B and beta-Actin. Levels of Ring1B were normalised to b-Actin. (A) One biological replicate and its 3 technical replicates (blots for all 3 biological replicates for Ring1B and b-Actin are provided as metadata), and (B) quantification of these data. Data analysed using the Student t test, error bars ± SD, each dot represents a single data point, p = *p < 0.05, ***p < 0.001; NMP-L cells were differentiated towards neural progenitors in control (DMSO only) or MEKi (PD032590, 3 μM)/DMSO conditions for 3 days (assessed on D6) in 3 independent differentiations, replicates 1,2,3,; (C, D) exposure to MEKi lead to increased expression of PAX6 in all three replicates detected by RT-qPCR (p = 0.0024); western blot of 2 technical replicates from each of 3 biological replicates (E, E’) confirm dephosphorylation of ERK1/2 with antibodies to p-ERK1/2 and compared with total ERK1/2 levels and show (F, F’) Ring1B and GAPDH protein levels were unaffected by loss of ERK signalling; (G) ChIP-qPCR detecting ERK2 occupancy at PAX6 and control loci during differentiation (NMP-L (D3), D5, and D6: black, dark grey, and light grey, respectively) (n = 3 independent experiments, error bars = SEM, * = p ≤ 0.05, t test, comparison between D3 and D6 is significant); (H and H’) ChIP-qPCR investigating ERK2 occupancy at the PAX6 locus on D5 and D6 of the differentiation protocol comparing untreated, DMSO, and MEKi-exposed samples (n = 3 independent experiment, error bars = SEM, no significant differences between treatments, t test, MEKi not enriched over IgG on D5 and D6). All underlying numerical data in this figure can be found in S7 Data. ChIP-qPCR, chromatin immunoprecipitation quantitative PCR; hESC, human ESC; MEKi, MEK inhibitor; NMP-L, NMP-like; NP, [file pbio.3000221.s004.pdf]

Supplementary Figures Semprich et al

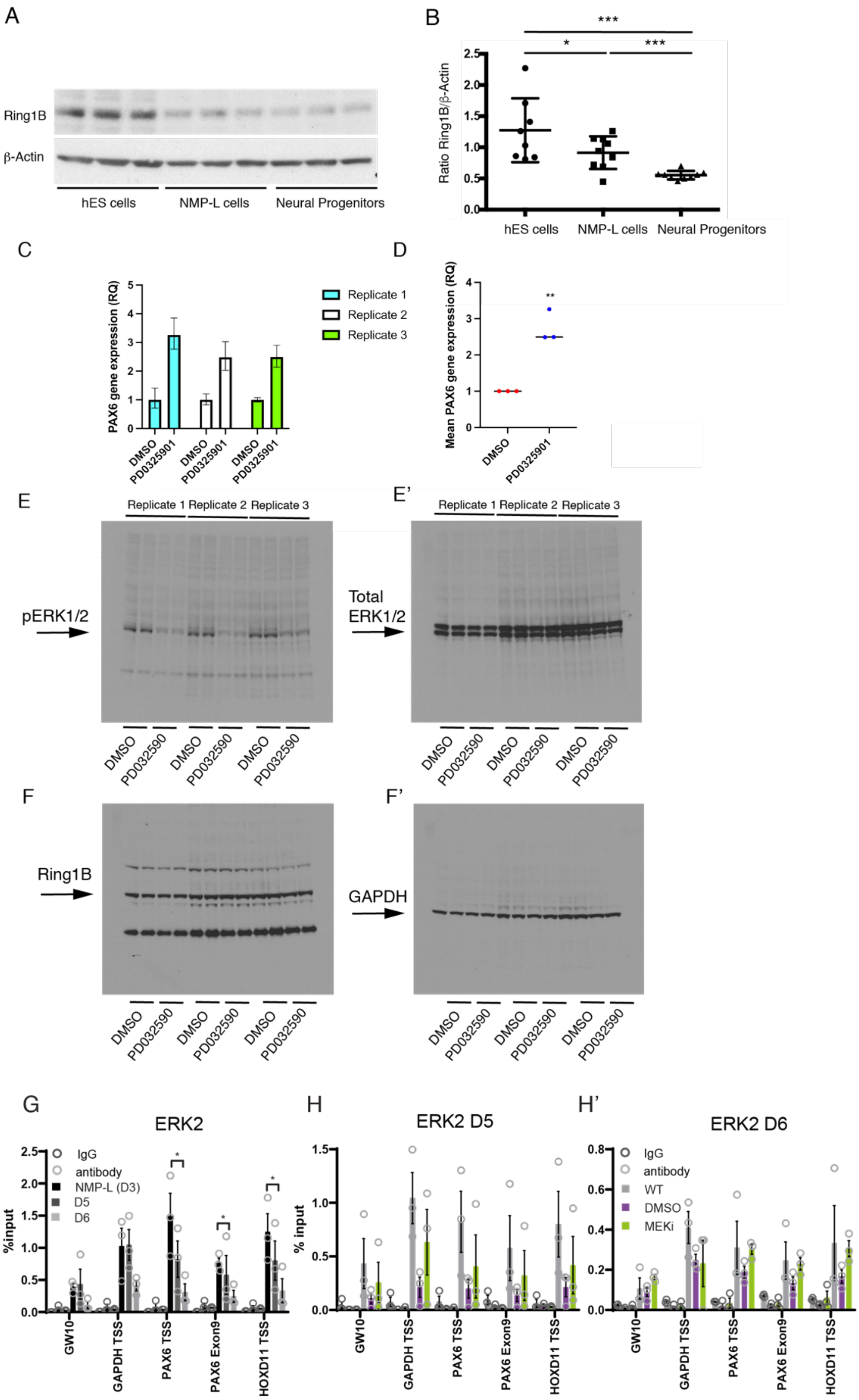

S4_Fig Analysis of global Ring1B levels and ERK2 occupancy during neural differentiation and following exposure to MEKi

To assess levels of Ring1B protein as hESCs differentiate into NMP-L cells and then NPs we collected protein lysates from 3 independent differentiations and ran Western blots, each with three technical replicates using

antibodies against Ring1B and beta-Actin. Levels of Ring1B were normalized to b-Actin; (A) one biological replicate and its 3 technical replicates (blots for all 3 biological replicates for Ring1B and b-Actin are provided as metadata), and (B) quantification of these data. Data analysed using the student T test, error bars ± SD, each dot represents a single data point, p = *p<0.05, ***p<0.001; NMP-L cells were differentiated towards neural progenitors in control (DMSO only) or MEKi (PD032590, 3 µM)/DMSO conditions for 3 days (assessed on D6) in 3 independent differentiations, replicates 1,2,3,; (C, D) exposure to MEKi lead to increased expression of *PAX6* in all three replicates detected by RTqPCR (p = 0.0024); Western blot of 2 technical replicates from each of 3 biological replicates (E,E') confirm dephosphorylation of ERK1/2 with antibodies to p-ERK1/2 and compared with total ERK1/2 levels; and show (F,F') Ring1B and GAPDH protein levels were unaffected by loss of ERK signalling; (G) ChIP-qPCR detecting ERK2 occupancy at *PAX6* and control loci during differentiation (NMP-L (D3), D5 and D6, black, dark grey and light grey respectively) (n = 3 independent experiments, error bars = SEM, * = p ≤ 0.05, T-test, comparison between D3 and D6 is significant); (H and H') ChIP-qPCR investigating ERK2 occupancy at the *PAX6* locus on D5 and D6 of the differentiation protocol comparing untreated, DMSO and MEKi exposed samples (n = 3 independent experiment, error bars = SEM, no significant differences between treatments, t-test, MEKi not enriched over IgG on D5 and D6).
