# Supplementary material for: ERK1/2 signalling dynamics promote neural differentiation by regulating chromatin accessibility and the polycomb repressive complex
Source: PLoS Biol. 2022 Dec 1;20(12):e3000221. doi: 10.1371/journal.pbio.3000221 (PMC9746999; doi:10.1371/journal.pbio.3000221)
Supplement: S5 Fig — (A) Differentiation protocol used to generate NMP-L (D3) cells and treatment regime with vehicle control DMSO or MEKi for 3 hours; (B-B’) representative western blot of cell lysates probed with antibodies against total (panERK1/2) and dual-phosphorylated-ERK1/2 (dpERK1/2) and LiCOR quantification data (n = 3 independent experiments, error bar = SEM, * p = < 0.05); (C-E) ChIP-qPCRs investigating Jarid2 and Ring1B occupancy and H3K27me3 levels at PAX6 and control regions in NMP-L (D3) cells treated with MEKi or DMSO for 3 hours (n = 3 individual experiments, bar = average, no significant differences between samples, t test), note low Jarid2 input, not enriched over IgG; (F-F”) transcription levels of PAX6, HOXD11, and JARID2 assessed by RT-qPCR in undifferentiated cell (hESCs), untreated, vehicle control (DMSO)-treated, or MEKi-treated NMP-L (D3) cells (n = 3 individual experiments, no significant differences between samples, t test). All underlying numerical data in this figure can be found in S9 Data. ChIP-qPCR, chromatin immunoprecipitation quantitative PCR; hESC, human ESC; IgG, immunoglobulin G; MEKi, MEK inhibitor; NMP-L, NMP-like; PRC, polycomb repressive complex; RT-qPCR, reverse transcription quantitative PCR. (PDF) [file pbio.3000221.s005.pdf]

Supplementary Figures Semprich et al

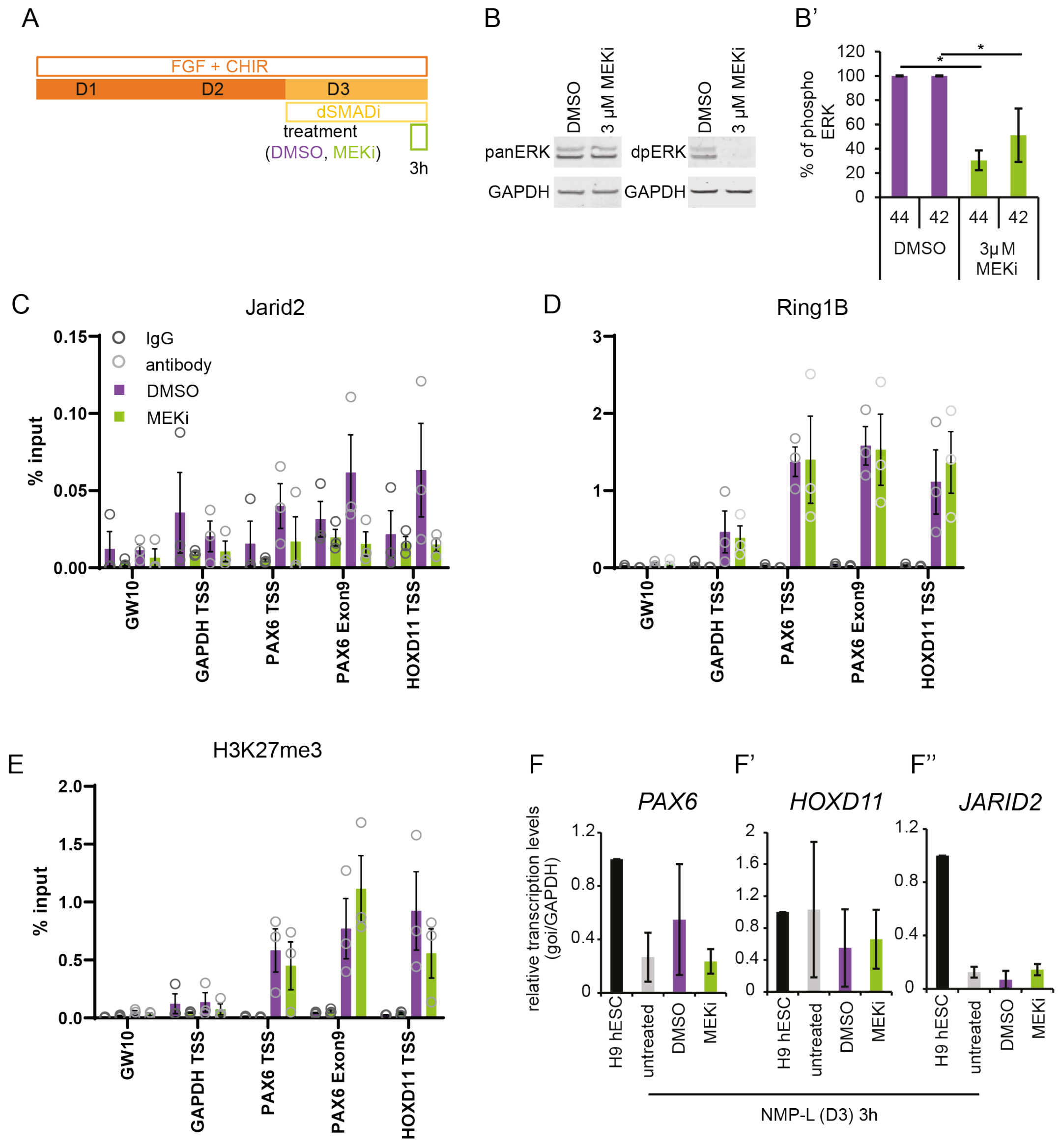

S5_Fig Brief ERK1/2 dephosphorylation in NMP-L cells does not alter PRC occupancy, H3K27me3 levels or transcription

(A) Differentiation protocol used to generate NMP-L (D3) cells and treatment regime with vehicle control DMSO or MEKi for 3h; (B-B') representative Western blot of cell lysates probed with antibodies against total (panERK1/2) and dual-phosphorylated-ERK1/2 (dpERK1/2) and LiCOR quantification data (n = 3 independent experiments, error bar = SEM, * p= <0.05); (C -E)) ChIP-qPCRs investigating Jarid2 and Ring1B occupancy and H3K27me3 levels at PAX6 and control regions in NMP-L (D3) cells treated with MEKi or DMSO for 3h (n = 3 individual experiments, bar = average, no significant differences between samples, t-test), note low Jarid2 input, not enriched over IgG; (F-F'') transcription levels of *PAX6, HOXD11,* and *JARID2* assessed by RTqPCR in undifferentiated cell (hESCs), untreated, vehicle control (DMSO) treated or MEKi treated NMP-L (D3) cells (n=3 individual experiments, no significant differences between samples, t-test).
