# Supplementary material for: ERK1/2 signalling dynamics promote neural differentiation by regulating chromatin accessibility and the polycomb repressive complex
Source: PLoS Biol. 2022 Dec 1;20(12):e3000221. doi: 10.1371/journal.pbio.3000221 (PMC9746999; doi:10.1371/journal.pbio.3000221)
Supplement: S6 Fig — (A and B) Western blot analysis of NMP-L (D3) protein extracts using phospho-specific antibodies against ERK1/2 and PKB alongside pan antibodies. (C and D) Quantification of band intensity shows no reduction in ERK phosphorylation but increased levels of PKB phosphorylation (n = 3 independent experiments error bars = SEM, * = p ≤ 0.05 or t test showed no significant difference). Underlying numerical data for western blots in this figure can be found in S11 Data. MEKi, MEK inhibitor; NMP-L, NMP-like. (PDF) [file pbio.3000221.s006.pdf]

Supplementary Figures Semprich et al

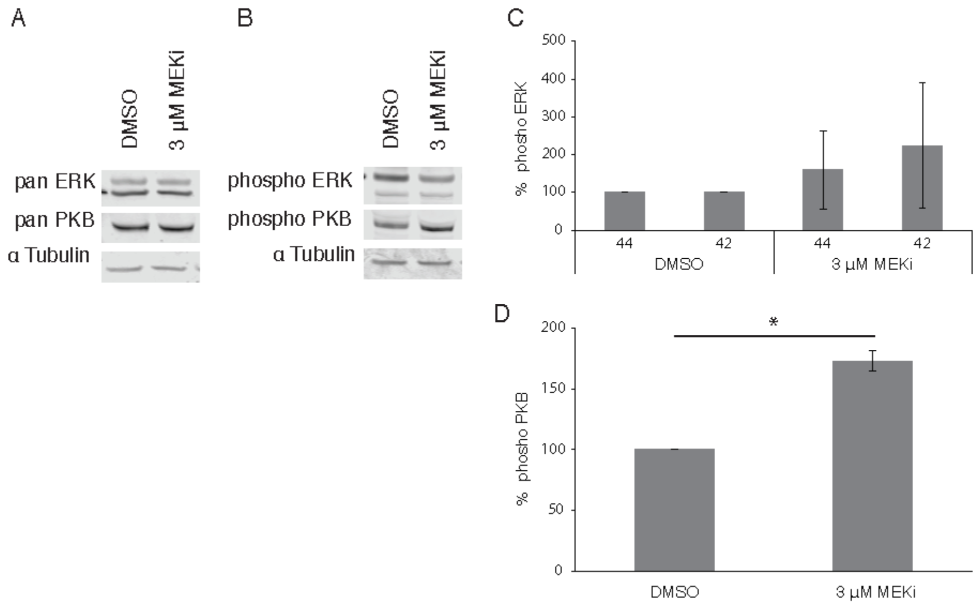

S6_Fig NMP-L cells cultured for 12h in MEKi no longer exhibit reduced ERK phosphorylation but have increased PKB phosphorylation levels (A and B) Western blot analysis of NMP-L (D3) protein extracts using phospho-specific antibodies against ERK1/2 and PKB alongside pan antibodies. (C and D) quantification of band intensity shows no reduction in ERK phosphorylation but increased levels of PKB phosphorylation (n = 3 independent experiments error bars = SEM, * = p ≤ 0.05 or t-test showed no significant difference).
