# Supplementary material for: ERK1/2 signalling dynamics promote neural differentiation by regulating chromatin accessibility and the polycomb repressive complex
Source: PLoS Biol. 2022 Dec 1;20(12):e3000221. doi: 10.1371/journal.pbio.3000221 (PMC9746999; doi:10.1371/journal.pbio.3000221)

Figure 1 C
protein extracted from 3 pooled embryos that were cultured in hanging drop prior to extraction
pan and dp blot with Tubulin as loading control, membranes scanned using LI-COR Odyssey System
membrane half marked 1b is showing signal for pan ERK
membrane half marked with 2b is showing signal for dp ERK
marker: BenchMark Pre-stained Protein Ladder, Invitrogen
pan ERK ab: # 9102 CST, dpERK ab: #9101 CST
tubulin ab: ab7291 abcam
secondary ab: Goat anti-Mouse IgG (H+L) Cross-Adsorbed Secondary Antibody, DyLight™ 800, #SA5-10176,
Goat anti-Rabbit IgG (H+L) Cross-Adsorbed Secondary Antibody, DyLight™ 680, #35569, both Thermo Fisher

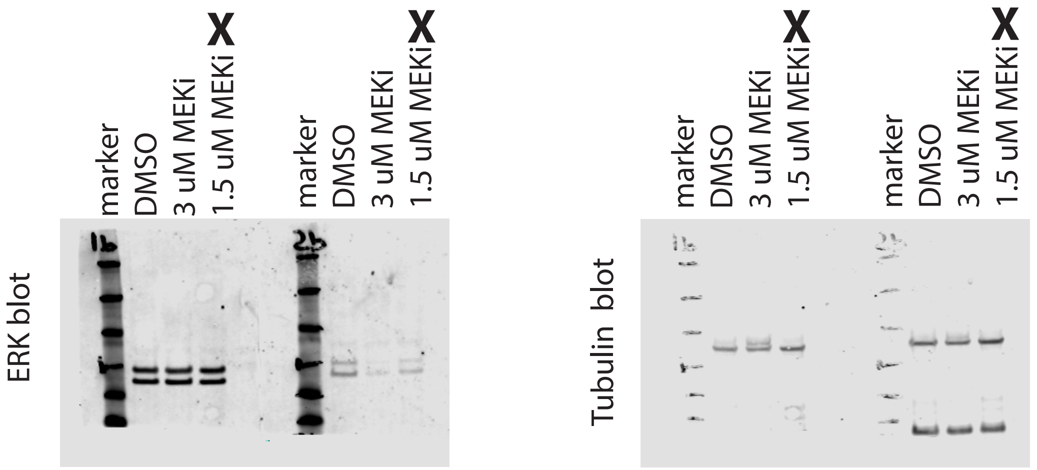

Figure 4B
protein extracted from human ESCs differentiated as indicated in paper prior to extraction
pan and dp blot with GAPDH as loading control, membranes scanned using LI-COR Odyssey System

marker: BenchMark Pre-stained Protein Ladder, Invitrogen
pan ERK ab: # 9102 CST, dpERK ab: #9101 CST
GAPDH ab: ab9484 abcam
secondary ab: Goat anti-Mouse IgG (H+L) Cross-Adsorbed Secondary Antibody, DyLight™ 800, #SA5-10176,
Goat anti-Rabbit IgG (H+L) Cross-Adsorbed Secondary Antibody, DyLight™ 680, #35569, both Thermo Fisher

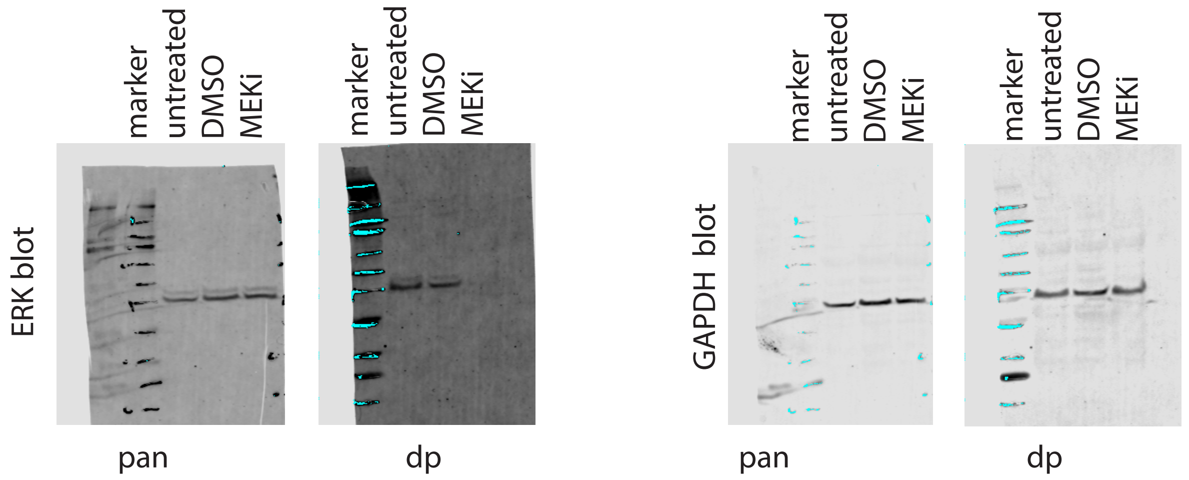

Figure 6B
protein extracted from human ESCs differentiated as indicated in paper prior to extraction
pan and dp blot with GAPDH as loading control, membranes scanned using LI-COR Odyssey System
left membrane is pan membrane, right membrane is dp membrane
dp ERK was rescanned as over exposed in the first scan and only saved as a zoom into ERK
on the same membrane ERK, PKB and Ezh2 were detected, for the Figure only ERK was used
marker: BenchMark Pre-stained Protein Ladder, Invitrogen
pan ERK ab: # 9102 CST, dpERK ab: #9101 CST
GAPDH ab: ab9484 abcam
secondary ab: Goat anti-Mouse IgG (H+L) Cross-Adsorbed Secondary Antibody, DyLight™ 800, #SA5-10176,
Goat anti-Rabbit IgG (H+L) Cross-Adsorbed Secondary Antibody, DyLight™ 680, #35569, both Thermo Fisher

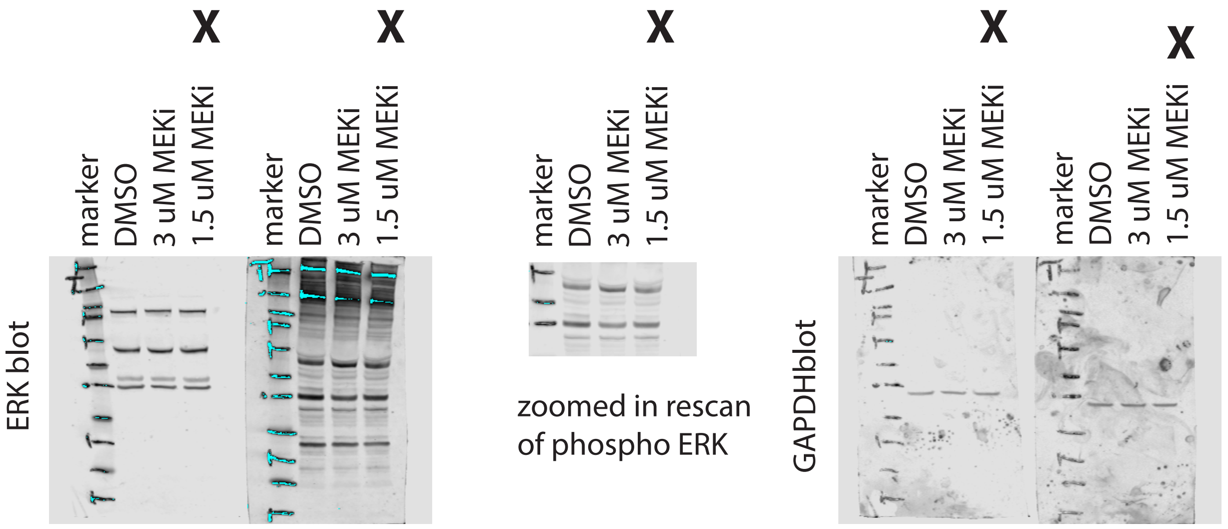

Figure S5 B
protein extracted from human ESCs differentiated as indicated in paper prior to extraction
pan and dp blot with GAPDH as loading control, membranes scanned using LI-COR Odyssey System
left membrane is 1h treatment whereas right membrane 3h treatment, for figure the 3h treatment was used.
on the same membrane ERK and PKB detected, for the Figure ERK and AKT were used
marker: BenchMark Pre-stained Protein Ladder, Invitrogen
pan ERK ab: # 9102 CST, dpERK ab: #9101 CST
GAPDH ab: ab9484 abcam
secondary ab: Goat anti-Mouse IgG (H+L) Cross-Adsorbed Secondary Antibody, DyLight™ 800, #SA5-10176,
Goat anti-Rabbit IgG (H+L) Cross-Adsorbed Secondary Antibody, DyLight™ 680, #35569, both Thermo Fisher

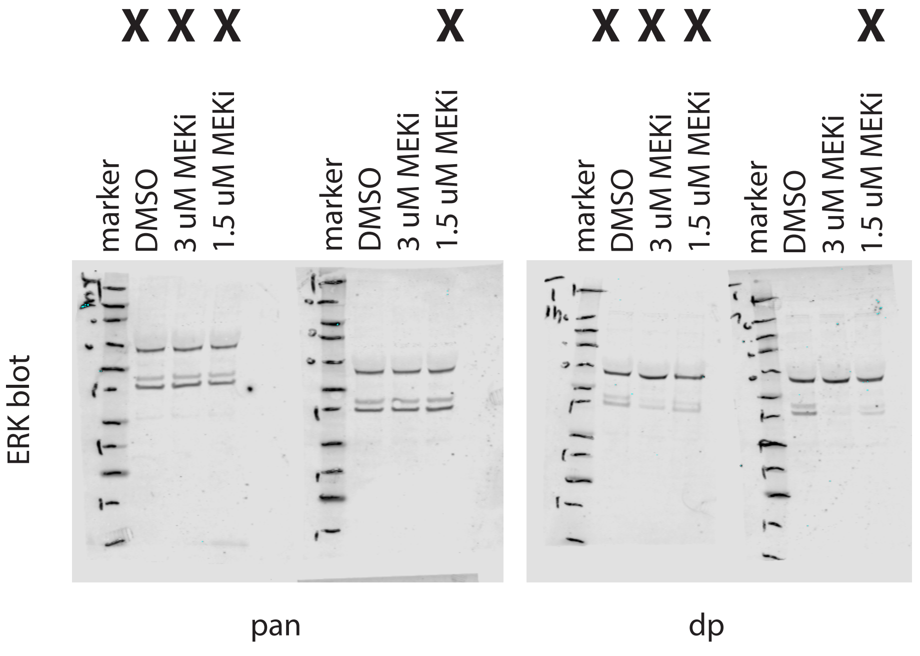

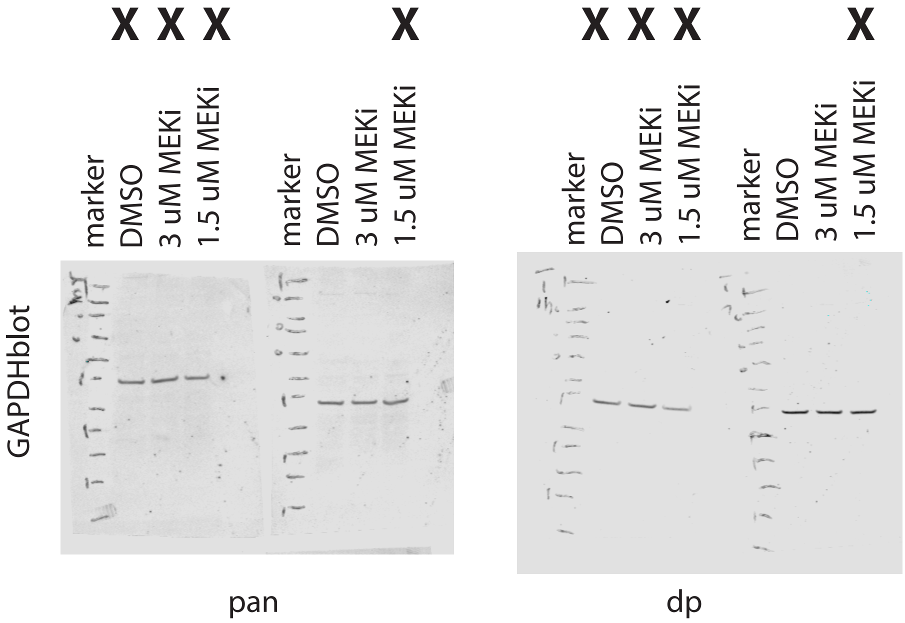

Figure S6 A and B

these are the same membranes as Figure 6B

protein extracted from human ESCs differentiated as indicated in paper prior to extraction

(A) pan and (B) dp blot with GAPDH as loading control, membranes scanned using LI-COR Odyssey System

left membrane is pan membrane, right membrane is dp membrane

dp ERK was rescanned as over exposed in the first scan and only saved as a zoom into ERK

p PKB was rescanned as over exposed in the first scan

on the same membrane ERK, Ezh2 and PKB detected, for the Figure ERK and PKB were used

marker: BenchMark Pre-stained Protein Ladder, Invitrogen

pan ERK ab: # 9102 CST, dpERK ab: #9101 CST, pan PKB ab #4691 CST, phospho PKB #4060 CST

GAPDH ab: ab9484 abcam

secondary ab: Goat anti-Mouse IgG (H+L) Cross-Adsorbed Secondary Antibody, DyLight™ 800, #SA5-10176, Goat anti-Rabbit IgG (H+L) Cross-Adsorbed Secondary Antibody, DyLight™ 680, #35569, both Thermo Fisher

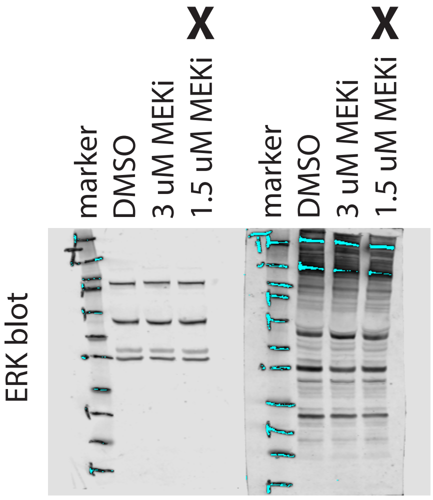

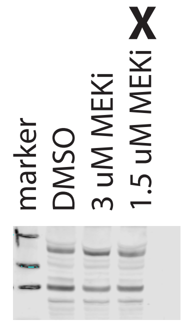

zoomed in rescan of phospho ERK

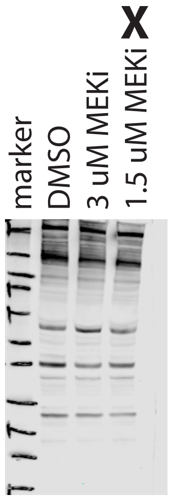

rescan of phospho PKB

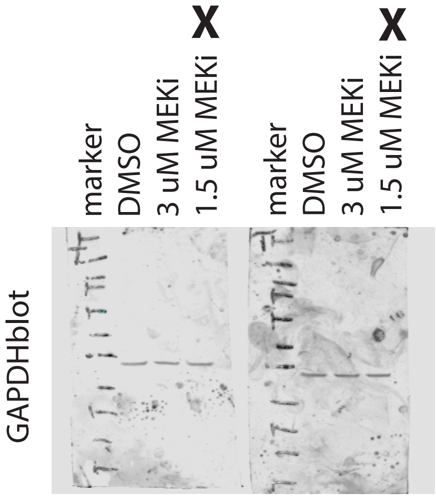

Figure S3B

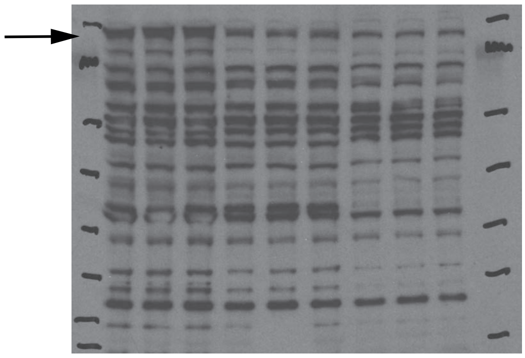

hES cells NMP-L cells Neural Progenitors

rabbit anti-Jarid2
Abcam ab181542

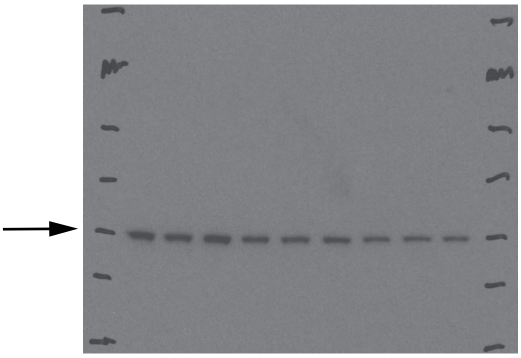

hES cells NMP-L cells Neural Progenitors

mouse anti-GAPDH
Abcam ab9484

Figure S4A

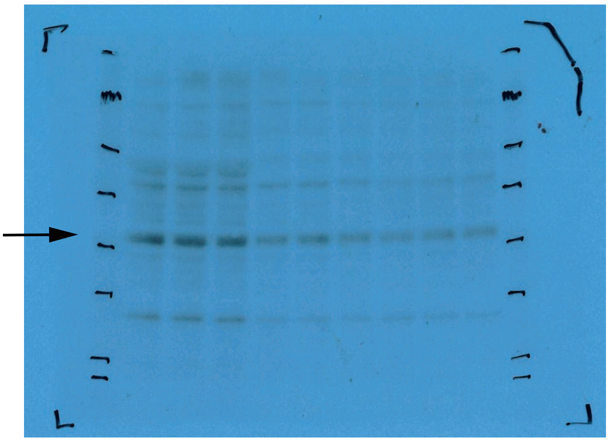

hES cells NMP-L cells Neural Progenitors

mouse anti-Ring1B
MBL D139-3

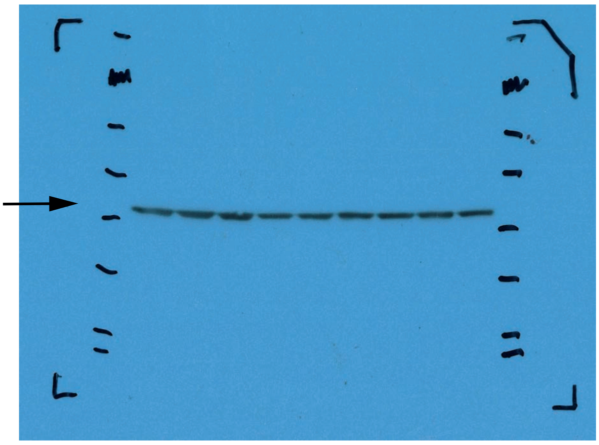

hES cells NMP-L cells Neural Progenitors

rabbit anti-beta-Actin
Abcam ab8227

Western blots for all three bioloical replicates (replicate 1 is shown in Figure S4A)

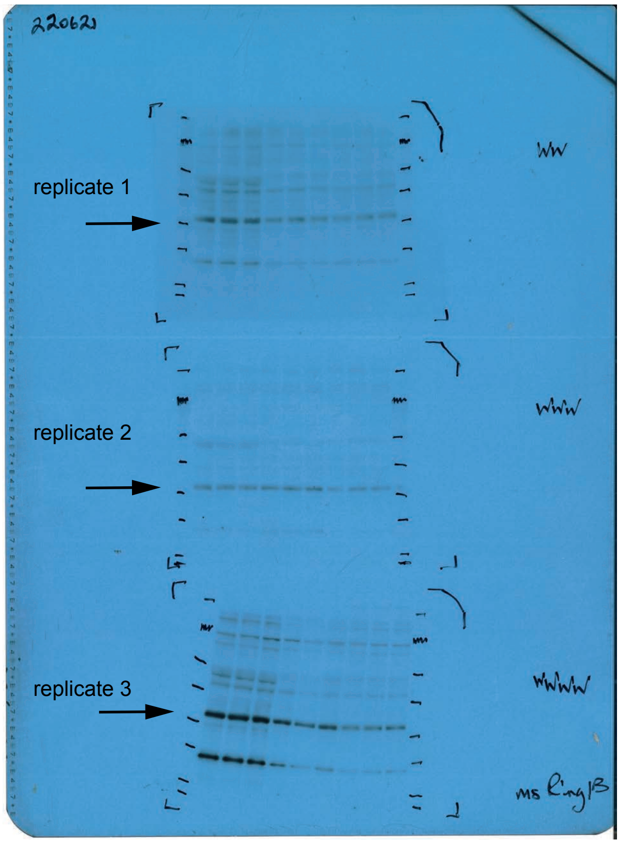

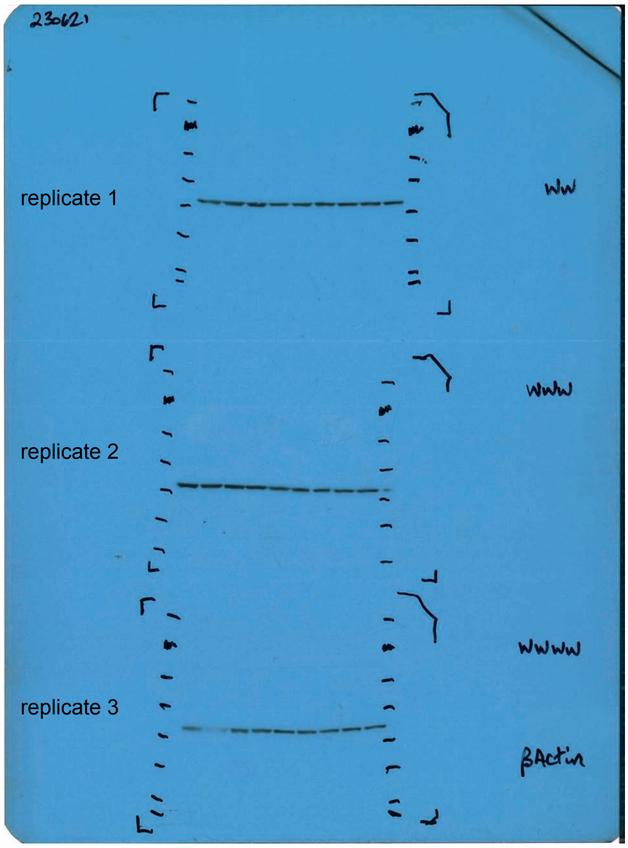

Supplement: S1 Raw Images — (PDF) [file pbio.3000221.s026.pdf]
